# Melittin‐Carrying Nanoparticle Suppress T Cell‐Driven Immunity in a Murine Allergic Dermatitis Model

**DOI:** 10.1002/advs.202204184

**Published:** 2023-01-13

**Authors:** Zheng Liu, Zhan Fan, Jinxin Liu, Jialu Wang, Mengli Xu, Xinlin Li, Yilun Xu, Yafang Lu, Chenlu Han, Zhihong Zhang

**Affiliations:** ^1^ Britton Chance Center and MOE Key Laboratory for Biomedical Photonics Wuhan National Laboratory for Optoelectronics Huazhong University of Science and Technology Wuhan Hubei 430074 China; ^2^ School of Biomedical Engineering Hainan University Haikou Hainan 570228 China

**Keywords:** allergic contact dermatitis, atopic dermatitis, dendritic cell modulation, lipid nanoparticles, melittin

## Abstract

Allergic contact dermatitis (ACD) and atopic dermatitis (AD) are the most common human skin disorders. Although corticosteroids have been widely used to treat ACD and AD, the side effects of corticosteroids encourage researchers to explore new immunoregulatory treatments. Here, an immunomodulatory approach based on lipid nanoparticles carrying *α*‐helical configurational melittin (*α*‐melittin‐NP) is developed to overcome T cell‐mediated inflammatory reactions in an oxazolone (OXA)‐induced contact hypersensitivity mouse model and OXA‐induced AD‐like mouse model. Intradermal injection of low‐dose *α*‐melittin‐NPs prevents the skin damage caused by melittin administration alone and efficiently targeted lymph nodes. Importantly, melittin and *α*‐melittin‐NPs restrain RelB activity in dendritic cells (DCs) and further suppresses dendritic cell activation and maturation in lymph nodes. Furthermore, low‐dose *α*‐melittin‐NPs leads to relief of antigen recognition‐induced effector T cell arrest in the dermis and inhibited allergen‐specific T cell proliferation and activation. Significantly, this approach successfully controls Th1‐type cytokine release in the ACD model and restricts Th2‐type cytokine and IgE release in the AD‐like model. Overall, intradermal delivery of low‐dose *α*‐melittin‐NPs efficiently elicits immunosuppression against T cell‐mediated immune reactions, providing a promising therapeutic strategy for treating skin disorders not restricted to the lesion region.

## Graphical abstract

A novel nanoimmunotherapy approach based on DC‐targeted *α*‐melittin‐ nanoparticles (NPs) is developed for treating allergic skin disease not restricted to the lesion region. Low‐dose *α*‐melittin‐NPs suppresses allergen‐stimulated DC activation and further inhibits allergen‐specific T cell proliferation and activation, leading to efficient control of both the Th1 and Th2 cell‐driven immune responses in allergic contact dermatitis and atopic dermatitis.

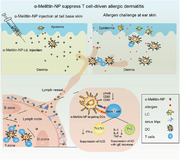

## 1 Introduction

Allergic contact dermatitis (ACD) and atopic dermatitis (AD) are common and widespread human diseases with increasing prevalence.^[^ ^1^, ^2^ ^]^ Most patients with ACD or AD experience a significant decrease in quality of life.^[^ ^3^ ^]^ The current treatment strategies include avoiding contact with allergens and topical corticosteroid application.^[^ ^4^, ^5^ ^]^ However, long‐term, improper usage of corticosteroids may lead to side effects, including skin thinning and atrophy.^[^ ^6^, ^7^ ^]^ Therefore, the exploration of new therapeutic approaches to cure ACD and AD is urgently needed. ACD and AD are mainly T cell‐mediated skin disorders,^[^ ^8^, ^9^ ^]^ and interferon gamma (IFN‐*γ*) and IL‐4 secreted from effector T cells are the primary cytokines that invoke the inflammatory reaction in ACD and AD.^[^ ^10^, ^11^ ^]^ Inhibiting the proliferation and activation of effector T cells is becoming an effective and promising strategy for treating ACD and AD.

Recently, nanoenabled immunomodulation, especially the induction of immune tolerance, has emerged as an active field of research in nanomedicine.^[^ ^12^ ^]^ Flexibly designed nanomaterials for suppressing specific immune responses offer promising alternatives for treating autoimmune diseases.^[^ ^13^, ^14^, ^15^, ^16^ ^]^ Among these strategies, the development of nanomaterials that target and reprogram dendritic cells (DCs) is the most promising strategy for inducing immune tolerance.^[^ ^12^ ^]^ Nanomaterials have also been widely used in cutaneous drug delivery to enhance drug stabilization, control drug release, and minimize compound toxicity. However, harnessing nanoimmunotherapy approaches to ameliorate ACD and AD is restricted to the topical delivery of medications (e.g., immunosuppressants, cAMP inhibitors, calcineurin inhibitors, or corticosteroids) to the lesion region, which control local skin inflammation. The development of a nanoimmunotherapy approach designed to induce peripheral immune tolerance to treat ACD and AD with few side effects is urgently needed. DCs in lymph nodes (LNs) are important targets for nanoenabled immunomodulation, and the costimulatory pathway in DCs is critical for Th1/Th2 differentiation of T cells.^[^ ^17^ ^]^ Applying nanomedicine to modulate the costimulatory pathway in DCs might provide a promising strategy for treating T cell‐driven skin disorders.

Bee venom is a traditional medicine that has been used to treat arthritis and liver cirrhosis.^[^ ^18^, ^19^, ^20^ ^]^ For some time, melittin, the major component of bee venom, has been recognized as an anti‐inflammatory drug to treat autoimmune diseases.^[^ ^21^ ^]^ However, intradermal injection of melittin was also reported to cause paw edema, and bee venom therapy was reported to induce monocyte activation, enhance Th1 cell‐mediated immunity and elicit an antitumor response.^[^ ^22^, ^23^, ^24^ ^]^ The contrasting function of melittin in immune modulation, namely, proinflammatory or anti‐inflammatory immunity, suggests that different doses of melittin or different delivery methods might exert opposite effects on the immune system. In addition, the side effects of melittin (e.g., erythrocyte lysis and skin damage) limit its application in vivo. Harnessing nanotechnology to deliver melittin for targeted regulation of the immune system while shielding its toxicity is expected to be a therapeutic approach with broad immunosuppressive effects.

Previously, we designed a high‐density lipoprotein‐mimicking peptide‐phospholipid scaffold (named *α*‐peptide‐NP).^[^ ^25^ ^]^ Loading melittin onto the scaffold to form ultrasmall *α*‐melittin‐loaded nanoparticles (*α*‐melittin‐NPs) reduced the cytotoxicity to red blood cells.^[^ ^26^ ^]^ Here, we developed a robust immunoregulatory approach based on an intradermal injection of *α*‐melittin‐NPs, which targets and modulates DCs in LNs. Low‐dose *α*‐melittin‐NPs prevent skin damage caused by melittin and efficiently control both Th1 and Th2 cell‐driven immune reactions in ACD and AD.

## 2 Results

### 2.1 Both Melittin and *α*‐Melittin‐NPs Efficiently Inhibit the Contact Hypersensitivity (CHS) Reaction

**Figure 1:**
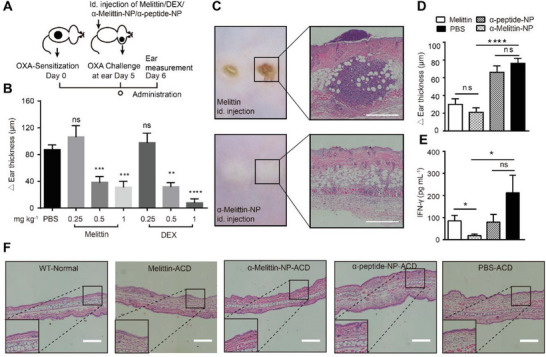
Melittin and melittin‐based lipid NPs inhibit ACD. A) Schematic illustration of *α*‐melittin‐NP therapy in a mouse model of ACD. C57BL/6 mice were sensitized with 2% OXA and challenged with 0.3% OXA 5 days after sensitization. B) Measurement of the increasing ear thickness in the ACD model with melittin or DEX intradermal injection at the tail base (*n* ≥ 5 mice per group, means ± SD). C) H&E staining of skin sections after the intradermal injection of melittin or *α*‐melittin‐NPs at the tail base. Scale bar, 400 µm. D) Measurement of ear swelling in the ACD model injected with PBS, melittin, *α*‐melittin‐NPs, and *α*‐peptide‐NPs at the tail base region (*n* ≥ 10 per group, means ± SEM). Data are representative of three trials. E) Measurement of serum IFN‐*γ* levels in the ACD model treated with PBS, melittin, *α*‐melittin‐NPs, and *α*‐peptide‐NPs (*n* = 7 mice per group, means ± SEM). F) H&E staining of ear sections from PBS‐, melittin‐, *α*‐melittin‐NP‐, and *α*‐peptide‐NP‐treated ACD model mice. Scale bar, 200 µm. *p < 0.05, **p < 0.01, ***p < 0.001, ****p < 0.0001, and ns, not significant; Statistical analysis was performed using a two‐tailed unpaired Student's *t*‐test.

To explore the effect of melittin on contact allergy, we used oxazolone (OXA) as a hapten to establish a mouse CHS model of ACD (**Figure** **1**A). Phosphate buffered saline (PBS), melittin, or dexamethasone (DEX) was intradermally injected at the tail base 30 min after OXA challenge in the ear. As shown in Figure 1B, similar to DEX, melittin significantly inhibited ear swelling in the CHS model in a dose‐dependent manner. Treatment with 0.5 mg kg^−1^ melittin (176 nmol kg^−1^) suppressed ear swelling by 55.8%, and treatment with 0.5 mg kg^−1^ DEX (1275 nmol kg^−1^) suppressed ear swelling by 63.3% compared to PBS treatment at 24 h after OXA challenge. Although melittin efficiently restrained the OXA‐induced CHS reaction, unfortunately, direct intradermal injection of melittin caused local inflammation, including excess leukocyte infiltration and epidermal damage (Figure 1C). Previously, *α*‐melittin‐NPs were reported to efficiently deliver melittin while preventing the blood toxicity of melittin.^[^ ^26^ ^]^ Compared to the intradermal injection of melittin at the tail base, *α*‐melittin‐NP treatment did not obviously damage the integration of the epidermal structure, and *α*‐melittin‐NPs attenuated the melittin‐induced excess accumulation of leukocytes at the injection site (Figure 1C). An additional ear swelling test also confirmed that *α*‐melittin‐NPs reduced 67% of the local damage caused by melittin at the injection site (Figure S1, Supporting Information). Previously, melittin was identified as an allergen that triggers mast cell degranulation and eventually leads to histamine release in allergic reaction. To explore whether *α*‐melittin‐NPs also attenuated melittin‐induced mast cell degranulation, we cultivated a murine mastocytoma cell line (P815) with different doses (0.01–5 µmol L^−1^) of *α*‐peptide‐NPs, *α*‐melittin‐NPs, and melittin. As shown in Figure S2 (Supporting Information), both compound 48/80 and melittin treatment significantly induced histamine release, while, there was no significant difference in histamine release between 5 µmol L^−1^ *α*‐melittin‐NPs‐ or *α*‐peptide‐NP‐treated group and the untreated group. These results indicated that *α*‐melittin‐NPs alleviated melittin‐induced local skin damage and mast cell degranulation.

Furthermore, we compared the immunoregulatory efficacy of *α*‐melittin‐NPs and melittin and confirmed that 176 nmol kg^−1^ *α*‐melittin‐NPs (concentration of melittin, 0.5 mg kg^−1^) also exerted strong curative effects on contact allergy that were similar to melittin. While the vehicle *α*‐peptide‐NPs alone did not exert curative effects (Figure 1D). Thus, an intradermal *α*‐melittin‐NP injection not only alleviated melittin‐induced local skin damage but also efficiently treated ACD. By detecting the levels of IFN‐*γ*, which provokes the immune reaction in CHS, we found that *α*‐melittin‐NP treatment almost completely suppressed serum IFN‐*γ* (> 90% suppression ratio) and that melittin treatment suppressed 59.4% of serum IFN‐*γ* compared with the levels in the PBS‐treated group (Figure 1E). In addition, the local levels of IL‐1*β*, IFN‐*γ*, IL‐4, and TNF‐*α* in the ear tissue were detected (Figure S3, Supporting Information), and the IL‐1*β* level was significantly altered by *α*‐melittin‐NPs, melittin, and DEX. The local levels of TNF‐*α*, IFN‐*γ*, and IL‐4 were not detected in the 0.3% OXA‐induced ACD model. In addition, hematoxylin and eosin (H&E) staining confirmed that both *α*‐melittin‐NP treatment and melittin treatment inhibited leukocyte infiltration and tissue damage in the CHS model (Figure 1F). Thus, our results revealed that *α*‐melittin‐NP treatment is superior to melittin treatment of ACD in controlling IFN‐*γ* production and in the safety of intradermal administration, and *α*‐melittin‐NPs might exert immunoregulatory effects by controlling IFN‐*γ* secretion.

### 2.2 Intradermal Injection of *α*‐Melittin‐NPs Targets LNs

**Figure 2:**
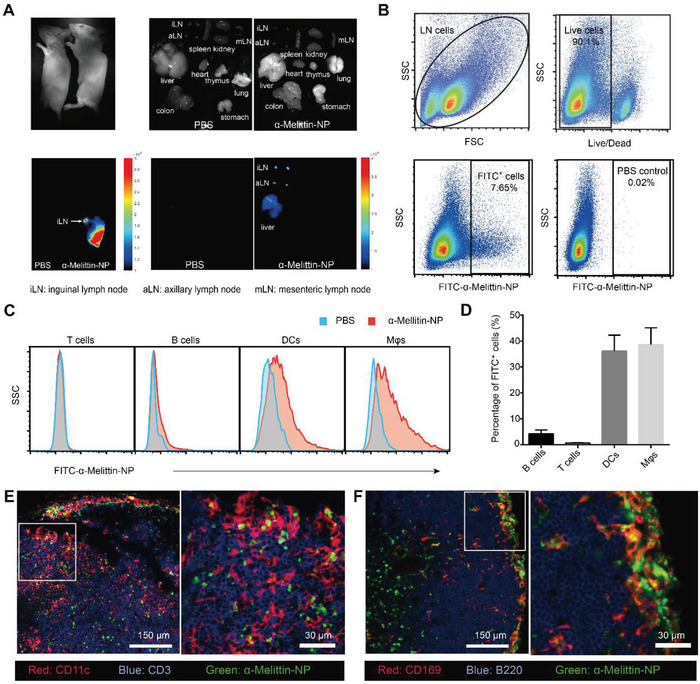
*α*‐Melittin‐NPs infiltrate the lymph nodes and target DCs and macrophages. A) Bright‐field (upper panel) and fluorescence microscopy images (lower panel) of organs and lymph nodes. Organs and lymph nodes were acquired at 12 h after *α*‐melittin‐NP (DiR‐BOA) (right panel) or PBS (left panel) injection at the tail base of albino C57BL/6 mice. Images are representative of three trials. The color‐coded scale for the fluorescence count is shown on the right. B) *α*‐Melittin‐NPs were conjugated to the fluorescent dye FITC, and FITC‐positive cells in the lymph nodes were analyzed using flow cytometry. C) Representative histograms indicating the mean fluorescence intensity (MFI) of FITC‐labeled *α*‐melittin‐NPs in DCs, M*φ*s, B cells, and T cells. D) Quantitative data for the FITC‐positive cells in (C), *n* = 4 mice per group, means ± SD. E,F) Representative images of immunofluorescence staining of LN sections from mice intradermally injected with *α*‐melittin‐NPs (FITC). Blue and red represent CD3^+^ T cells and CD11c^+^ DCs, respectively, and green represents FITC‐labeled *α*‐melittin‐NPs in (E). Red represents CD169^+^ macrophages, and blue represents B220^+^ B cells in (F). Scale bars, 150 µm (left panel) and 30 µm (right panel).

To further study the immunoregulatory mechanism of *α*‐melittin‐NPs, we used an in‐house whole‐body fluorescence imaging system to detect the fluorescent signal of *α*‐melittin‐NPs loaded with DiR‐BOA in mouse organs.^[^ ^27^ ^]^ As shown in **Figure** **2**A, *α*‐melittin‐NPs (DiR‐BOA) accumulated in the inguinal LNs, axillary LNs, and liver at 12 h postinjection but were detected at low levels or not detected in other organs. The semiquantification of the *α*‐melittin‐NP distribution confirmed that *α*‐melittin‐NPs are mainly restricted to the injected skin and the draining LNs within 24 h after *α*‐melittin‐NPs injection (Figure S4, Supporting Information). Next, *α*‐melittin was labeled with fluorescein isothiocyanate (FITC) to evaluate the uptake capacity of *α*‐melittin‐NPs in T cells (CD19^−^CD3^+^), B cells (CD19^+^CD3^−^), neutrophils (CD11b^+^Ly‐6G^+^), DCs (MHC‐II^+^CD11c^+^), and macrophages (M*φ*s) (CD11b^hi^F4/80^+^) (Figure 2B; and Figures S5and S6, Supporting Information). As seen in Figure 2C, the mean fluorescence intensity (MFI) of FITC in DCs and M*φ*s, but not in T cells and B cells, was significantly increased after the FITC^+^ *α*‐melittin‐NP intradermal injection. The data in Figure 2D further confirmed that DCs and M*φ*s exhibited a higher *α*‐melittin‐NP uptake capacity (36.2% in DCs and 38.6% in M*φ*s) than B cells and T cells (4.3% in B cells and 0.6% in T cells). In addition, the accumulation of *α*‐melittin‐NPs in DCs, M*φ*s, and neutrophils was not significantly different between inflammatory and noninflammatory contexts (Figure S5, Supporting Information). Furthermore, confocal imaging of LN sections at 24 h after the intradermal injection of *α*‐melittin‐NPs confirmed that FITC^+^ *α*‐melittin‐NPs were mainly captured by CD11c^+^ cells in the T cell zone and CD169^+^ cells in the capsular region (Figure 2E,F). None obvious FITC signal was detected in CD3^+^ cells in the T cell zone or B220^+^ cells in the B cell zone. Collectively, these results indicated that an intradermal injection of *α*‐melittin‐NPs efficiently targeted LNs and that *α*‐melittin‐NPs might inhibit the CHS reaction by modulating allergen presentation in DCs/M*φ*s in LNs.

### 2.3 *α*‐Melittin‐NPs Inhibit Allergen‐Induced DC Maturation

**Figure 3:**
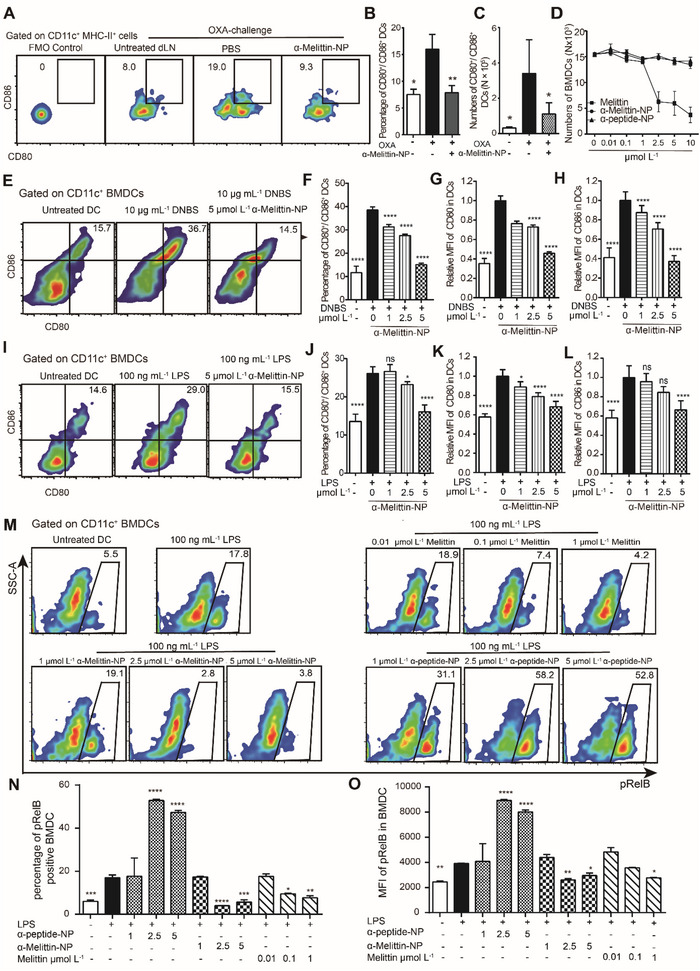
*α*‐Melittin‐NPs suppress DC activation and maturation both in vivo and in vitro. A) Representative flow cytometry plots for the CD80/CD86 dual‐positive DC proportion of dLN cells in the ACD model. B) Histogram showing the quantitative analysis of the data presented in (A) (*n* = 6 mice per group, means ± SD). dLNs were harvested from OXA‐challenged mice 24 h after the intradermal injection of *α*‐melittin‐NPs or PBS. C) Absolute cell count of the CD80/CD86 dual‐positive DC proportion among dLN cells. D) Quantification of the toxicity of melittin, *α*‐peptide‐NPs, and *α*‐melittin‐NPs toward BMDCs. BMDC viability was evaluated at 1 h after an incubation with 0, 0.01, 0.1, 1, 2.5, 5, or 10 µmol L^−1^ melittin, *α*‐peptide‐NPs, or *α*‐melittin‐NPs. E) Representative flow cytometry plots for CD80/CD86 dual‐positive cells in DNBS‐stimulated BMDCs cocultured with *α*‐melittin‐NPs in vitro. F) Quantitative analysis of CD80 and CD86 dual‐positive DCs in (C). G,H) Quantitative analysis of CD80 G) and CD86 H) MFIs in DNBS‐stimulated DCs (*n* = 5 mice per group, means ± SD). I) Representative flow cytometry plots for CD80/CD86 dual‐positive cells in LPS‐stimulated BMDCs cocultured with *α*‐melittin‐NPs in vitro. J) Quantitative analysis of the CD80 and CD86 dual‐positive DCs in (I). K–L) Quantitative analysis of CD80 K) and CD86 L) MFIs in LPS‐stimulated DCs (*n* ≥ 5 mice per group, means ± SD). M–O) Flow cytometry analysis of the NF‐*κ*B signaling molecule pRelB in BMDCs. M) Representative flow cytometry plots of LPS‐stimulated BMDCs cocultured with melittin, *α*‐melittin‐NPs, and *α*‐peptide‐NPs. N) Quantification of the percentage of pRelB‐positive BMDCs (*n* ≥ 6 mice per group, means ± SD). Quantitative analysis of the MFI of pRelB O) in LPS‐stimulated DCs. *p < 0.05, **p < 0.01, and ****p < 0.0001. Statistical analysis was performed using one‐way ANOVA followed by Bonferroni's post hoc test in (B,C), (F–H), (J–L), and (N–O). The data were pooled from two or three independent experiments (B–D, F–H, J–L, and M–O).

As DCs and hapten‐sensitized T cells in LNs mediate allergen recognition in CHS, we next explored DC maturation and T cell activation after *α*‐melittin‐NP entry into skin‐draining LNs (dLNs). Here, the CD80^+^/CD86^+^ DC (MHC‐II^+^CD11c^hi^) population in the dLNs increased from 6.4% (untreated mice) to 16.0% at 24 h after hapten challenge, indicating that hapten challenge effectively activated DCs in the dLNs (**Figure** **3**A–C). After treatment with *α*‐melittin‐NPs, the percentage of CD80^+^/CD86^+^ DCs in the LNs was maintained at 7.9%, which was not significantly different from that in untreated mice. The number of CD80^+^/CD86^+^ DCs in the dLNs was also significantly decreased in the *α*‐melittin‐NP group (1.1 × 10^4^ per dLN) compared with the PBS group (3.4 × 10^4^ per dLN). Although Langerhans cells (LCs) and dermal DCs/macrophages also phagocytosed *α*‐melittin‐NPs locally (Figure S7, Supporting Information), the results presented in Figure S8 (Supporting Information) indicated that *α*‐melittin‐NPs did not alter the migration of CD207^+^ LCs and CD207^−^ DCs to dLNs in FITC‐challenged skin. Thus, administration of *α*‐melittin‐NPs suppressed DC maturation in the dLNs in the OXA‐induced CHS model.

To investigate the modulatory effect of *α*‐melittin‐NPs on allergen‐stimulated DC activation and maturation, we first explored the potential toxicity of melittin and *α*‐melittin‐NPs. As shown in Figure 3D, both *α*‐peptide‐NP and *α*‐melittin‐NP were not toxic to bone marrow (BM)‐derived dendritic cell (BMDC) at concentrations ranging from 0.01 to 10 µmol L^−1^. When the concentration of melittin reached 2.5 µmol L^−1^, melittin was toxic to BMDCs. As a higher concentration (≥2.5 µmol L^−1^) of melittin is toxic to BMDCs, the maximum concentration of melittin we used for the BMDC activation experiment was 1 µmol L^−1^. Next, we chose dinitrobenzene sulfonic acid (DNBS) as the model allergen to stimulate BMDC activation (Figure 3E–H). After 10 µg mL^−1^ DNBS stimulation, the proportion of CD80^+^CD86^+^ BMDCs increased from 11.7% to 38.5%; this population was termed DNBS‐DCs. When DNBS and *α*‐melittin‐NPs (1, 2.5, or 5 µmol L^−1^) were simultaneously added to BMDCs, the proportion of CD80^+^CD86^+^ BMDCs was 31.3% (1 µmol L^−1^), 27.6% (2.5 µmol L^−1^), or 15.1% (5 µmol L^−1^); these proportions were significantly lower than those of DNBS‐stimulated BMDCs incubated without *α*‐melittin‐NPs. The MFI data for DNBS‐stimulated BMDCs showed that *α*‐melittin‐NPs downregulated allergen‐induced CD80 and CD86 expression in BMDCs. Furthermore, the data in Figure 3I–L; and Figures S9–S10 (Supporting Information) further confirmed that *α*‐melittin‐NP treatment significantly inhibited the lipopolysaccharide (LPS)‐induced differentiation of CD80^+^CD86^+^ BMDCs and CD80^+^CD86^+^ BM‐derived macrophages (BMDMs). Although the vehicle nanoparticle induced DC's maturation, this effect did not offset the inhibition of *α*‐melittin‐NP. We speculate that melittin in *α*‐melittin‐NP inhibits *α*‐peptide‐NP induced‐DC maturation. Thus, these results support the conclusion that *α*‐melittin‐NPs strongly modulate DC and M*φ*s activation in immune reactions.

### 2.4 Melittin and *α*‐Melittin‐NPs Restrain RelB Activity During DC Maturation

Next, we further explored how melittin, melittin‐based NPs and vehicle NPs regulate the maturation and activation of DCs. Here, we detected RelA and RelB activation in the nuclear factor‐kB (NF‐*κ*B) signaling pathway in BMDCs (Figure 3M–O; and Figure S11, Supporting Information). Sixty minutes after 100 ng mL^−1^ LPS treatment, the proportion of pRelB‐positive DCs increased from 5.45% to 17.8%, the MFIs of pRelB increased from 2444.3 to 3907.2. The increase in pRelB levels in BMDCs indicated the activation of the NF‐*κ*B signaling pathway during LPS‐stimulated DC maturation. A dose—response experiment of melittin (0.01, 0.1, and 1 µmol L^−1^), *α*‐melittin‐NPs (1, 2.5, and 5 µmol L^−1^) and *α*‐peptide‐NPs (1, 2.5, and 5 µmol L^−1^) was conducted to explore their effects on the phosphorylation of RelB during LPS‐stimulated DC maturation (Figure 3M–O). We found that 2.5 and 5 µmol L^−1^ *α*‐melittin‐NPs significantly reduced the levels of CD80/CD86 and pRelB in BMDCs. The 1 µmol L^−1^ *α*‐peptide‐NP treatment exerted little inhibitory effect on RelB activation, while the 2.5 and 5 µmol L^−1^ *α*‐peptide‐NP‐treated groups exhibited strong activation of CD80/CD86 and pRelB in BMDCs. Melittin (1 µmol L^−1^) effectively inhibited the phosphorylation of RelB in BMDCs. Interestingly, 1 µmol L^−1^ melittin significantly reduced the phosphorylation of RelA at 15 min with melittin treatment, however, this reduction is recovered at 60 min with melittin treatment. Thus, melittin and *α*‐melittin‐NPs restraining RelB activity in a dose‐dependent manner during DC maturation.

### 2.5 *α*‐Melittin‐NPs Inhibit CD8^+^ T Cell Proliferation and Activation in the CHS Model

**Figure 4:**
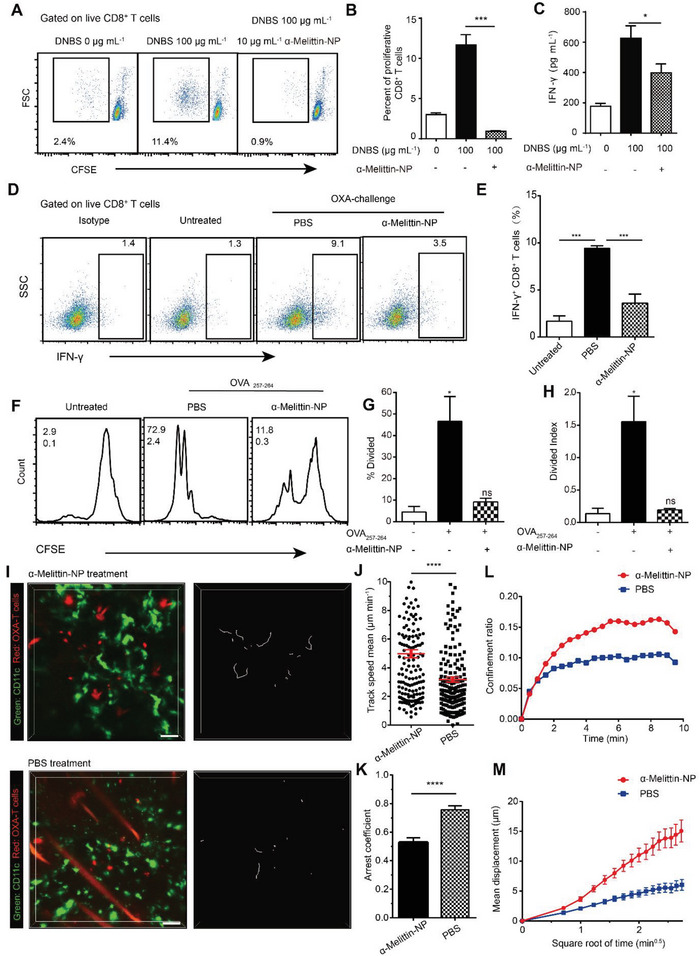
*α*‐Melittin‐NPs inhibit CD8^+^ T cell proliferation and activation. A) Representative image of DNFB‐sensitized CD8^+^ T cell proliferation after DNBS stimulation. CFSE‐labeled DNFB‐sensitized LN cells were stimulated with DNBS, and the proliferation of DNFB‐sensitized T cells was evaluated by measuring the CFSE signal decay using flow cytometry. B) Quantification of the percentage of proliferating DNFB‐sensitized T cells (*n* ≥ 8 samples per group, means ± SEM). C) *α*‐Melittin‐NP treatment reduced the IFN‐*γ* concentration in the supernatant of DNFB‐sensitized LN cells (*n* ≥ 8 samples per group, means ± SEM). D,E) Representative flow cytometry plots D) and histogram showing the quantitative analysis E) of IFN‐*γ*‐positive dLN CD8^+^ T cells in the OXA‐induced CHS model. F) Representative flow cytometry plots of OVA_257‐264_‐stimulated CD8^+^ T cell proliferation. Numbers in the upper left indicate the percentage of divided cells (% Divided), and numbers in the lower left are the average number of cell divisions (Div. Index). G) Histogram showing the quantification of the percentage of divided cells (% Divided) and H) average number of cell divisions (Div. Index). I) Representative intravital imaging of DCs and OXA‐sensitized T cells in the dermis of CHS mice treated with *α*‐melittin‐NPs or PBS; scale bar, 50 µm. The dLNs from OXA‐sensitized mice were harvested on day 4 to purify OXA‐sensitized T cells. The OXA‐sensitized T cells were adoptively transferred into CD11c^Venus^ mice. J–M) Motility parameters of CD8^+^ T cells in the dermis of mice treated with *α*‐melittin‐NPs (*n* > 158 cells) or PBS (*n* > 161 cells) at 18–24 h after OXA challenge. Track of the mean speed J), arrest coefficient K), confinement ratio L), and mean displacement M). The arrest coefficient ranges from 0 to 1, and a value close to 1 indicates that cells have stopped moving. *p < 0.05, **p < 0.01, ***p < 0.001, and ****p < 0.0001; Statistical analysis was performed using a two‐tailed unpaired Student's *t*‐test in (B,C), (E), (G,H), and (J,K). The data are representative of two or three independent experiments (A, F, and I) or were pooled from two or three independent experiments (B–E, G,H, and J–M).

Furthermore, we explored the effects of *α*‐melittin‐NPs on antigen‐specific T cell proliferation and activation. Here, we performed a DNBS‐induced lymphocyte proliferation assay to investigate the effects of *α*‐melittin‐NPs on hapten‐induced CD8^+^ T cell activation and proliferation. Five days after mice were sensitized with 2,4‐dinitrofluorobenzene (DNFB) on the abdomen, the dLNs were collected and restimulated with DNBS in vitro. As shown in **Figure** **4**A,B, the rate of DNBS‐stimulated CD8^+^ T cell proliferation in the control group was 11.7%, and the proliferation rate declined to 0.9% after treatment with *α*‐melittin‐NPs. Furthermore, the IFN‐*γ* concentration in cultured dLN cell supernatants also declined from 626.4 to 398.5 pg mL^−1^ after *α*‐melittin‐NP treatment (Figure 4C). These results indicated that *α*‐melittin‐NPs substantially inhibited DNBS‐stimulated CD8^+^ T cell proliferation and controlled IFN‐*γ* secretion in T cells.

Next, we detected T cell activation after *α*‐melittin‐NP entry into the dLNs. As shown in Figure 4D,E, the percentage of IFN‐*γ* ^+^ CD8^+^ T cells in the LNs of untreated mice was 1.3%. Twenty‐four hours after hapten challenge, the IFN‐*γ* ^+^ CD8^+^ T cells in the dLNs of untreated mice increased to 9.1%, indicating that hapten challenge effectively activated CD8^+^ T cells in the dLNs. After treatment with *α*‐melittin‐NPs in mice, the percentage of IFN‐*γ* ^+^ CD8^+^ T cells in the dLNs was maintained at 3.5%. These results indicated that *α*‐melittin‐NPs strongly inhibited DNBS‐stimulated CD8^+^ T cell activation. We further clarified the potential inhibitory effect of *α*‐melittin‐NPs on CD8^+^ T cell proliferation in vitro by investigating the effect of *α*‐melittin‐NPs on ovalbumin (OVA)‐induced T cell‐specific proliferation. Here, OVA‐specific T cell proliferation assays were performed to determine whether *α*‐melittin‐NPs also suppressed T cell proliferation upon stimulation with other antigens. As shown in Figure 4F,G, the percentage of dividing CD8^+^ T cells detected upon OVA_257‐264_ stimulation was 9.2% after *α*‐melittin‐NP treatment, which was significantly lower than that observed after PBS treatment (46.5%). The cell division number (Div. Index) of CD8^+^ T cells also showed a similar decline upon *α*‐melittin‐NP treatment (Figure 4H).

To further confirm whether *α*‐melittin‐NP suppresses T cell's proliferation and activation through DC modulation, DCs were first incubated with *α*‐melittin‐NP. At 24 h after culture, DCs were harvested and washed twice before coculture with T cells. As seen in Figure S12 (Supporting Information), OVA‐specific CD4 or CD8 T cell proliferation assays were significantly inhibited in the *α*‐melittin‐NP‐treated DC group. In Figure S12A–C (Supporting Information), the percentage of divided CD4^+^ T cells stimulated by OVA_323‐339_ was 8.9% after *α*‐melittin‐NP treatment, which was significantly lower than that observed after PBS treatment (22.0%) or *α*‐peptide‐NP treatment (29.0%). In Figure S12D–F (Supporting Information), the percentage of divided CD8^+^ T cells stimulated by OVA_257‐264_ was 37.6% after *α*‐melittin‐NP treatment, which was significantly lower than that observed after PBS treatment (55.7%) or *α*‐peptide‐NP treatment (56.3%). The cell division number (Div. Index) of CD8^+^ T cells or CD4^+^ T cells also showed a similar decline upon *α*‐melittin‐NP treatment (Figures S12C and S12F, Supporting Information). Thus, *α*‐melittin‐NPs efficiently restricted antigen‐specific T‐cell activation and proliferation via DC modulation.

As antigen‐specific T cells encountering dermal antigen‐presenting cells (APCs) and recognizing antigens in the skin are critical events in T cell‐mediated CHS,^[^ ^28^, ^29^ ^]^ we then explored the effect of *α*‐melittin‐NPs on antigen recognition between hapten‐specific T cells and APCs in the dermis through intravital imaging of mouse skin.^[^ ^30^, ^31^, ^32^ ^]^ Intravital imaging data in Figure 4I confirmed that *α*‐melittin‐NP treatment‐induced hapten‐specific T cells displayed more unrestricted behavior with a higher migration speed (5 µm min^−1^) and lower arrest coefficient (value: 0.5) when compared to PBS treatment. The mean square displacement versus time (MSD‐T) data further confirmed that T cells in the *α*‐melittin‐NP‐treated group were prone to more diffuse movement, with a higher confinement ratio than that of T cells in the PBS‐treated control group (Figure 4J–M). These parameters together suggested that *α*‐melittin‐NP treatment reduced antigen recognition in effector T cells arrest in the dermis. Effector T cells were reported to reduce their migration and mobility and stably interact with APCs upon antigen challenge and recognition, which promoted in situ activation of effector T cells with IFN‐*γ* upregulation.^[^ ^28^, ^33^, ^34^ ^]^ Thus, the *α*‐melittin‐NP treatment‐mediated decrease in effector T cell arrest might further inhibit the in situ activation of effector T cells along with downregulated IFN‐*γ* expression, which is consistent with the decrease in the IFN‐*γ* concentration detected in the CHS model after *α*‐melittin‐NP treatment.

### 2.6 *α*‐Melittin‐NPs Suppress the Immune Reaction in an OXA‐Induced AD‐Like Model

**Figure 5:**
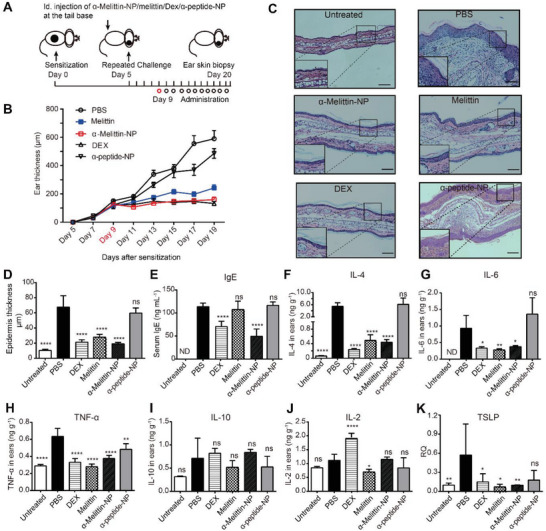
*α*‐Melittin‐NPs alleviate OXA‐induced AD. A) Treatment scheme for OXA‐induced AD. C57BL/6 mice were sensitized with 2% OXA at day 0. The sensitized mice were rechallenged with 0.1% OXA every other day (challenges on days 5, 7, 9, 11, 13, 15, 17, and 19 after sensitization). PBS, *α*‐melittin‐NPs, melittin, *α*‐peptide‐NPs, or DEX was administered from day 9 to day 19 (*n* = 8 mice per group). B) Quantification of increasing ear thickness in the AD model treated with PBS, melittin, *α*‐melittin‐NPs, *α*‐peptide‐NPs, or DEX. C‐E) H&E staining of ear sections C), epidermal thickness D), and serum IgE levels E) in the AD model treated with PBS, melittin, *α*‐melittin‐NPs, *α*‐peptide‐NPs, or DEX. The ears of AD model mice from the PBS‐, *α*‐melittin‐NP‐, melittin‐, *α*‐peptide‐NP‐, and DEX‐treated groups were harvested (*n* = 8 mice per group). Total protein and mRNA were extracted to assess Th2 cytokine levels and TLSP expression. F) Quantification of the IL‐4 concentration in each group. G) Quantification of the IL‐6 concentration in each group. H) Quantification of the TNF‐*α* concentration in each group. I) Quantification of the IL‐10 concentration in each group. J) Quantification of the IL‐2 concentration in each group. K) Quantification of the relative TSLP mRNA expression in each group. RQ, relative quantification. ND, not detected. Error bars indicate SD in (D) and (F–K), and SEM in (E); *p < 0.05, **p < 0.01, ***p < 0.001, ****p < 0.0001, and ns, not significant; Statistical analysis was performed using one‐way ANOVA followed by Bonferroni's post hoc test. Scale bar, 100 µm. The data are representative of two independent experiments C) or were pooled from two independent experiments (B and D–K).

Next, we explored the therapeutic effect of *α*‐melittin‐NPs on AD. Here, an OXA‐induced AD‐like mouse model was performed (**Figure** **5**A). As shown in Figure 5B, *α*‐melittin‐NPs administration achieved treatment efficiency with 82.1% suppression of ear swelling in the AD‐like model. After applying melittin, DEX administration achieved treatment efficiency by suppressing 63.6% and 84.6% of ear swelling in the AD‐like model, while *α*‐peptide‐NP administration showed no significant curative effects. H&E staining of ear sections showed that *α*‐melittin‐NP, melittin, and DEX treatments inhibited leukocyte infiltration and epidermal hyperplasia development in the AD‐like model (Figure 5C,D). Furthermore, the results in Figure 5E confirmed that *α*‐melittin‐NP treatment efficiently controlled the serum IgE level (56.6% decrease), similar to DEX treatment. This decrease was not observed in melittin treatment, suggesting that the intradermal injection of *α*‐melittin‐NPs was superior to melittin treatment in treating AD.

Th2 cell‐mediated immunity in AD is reported to be critical for allergic reactions in the skin. Having confirmed that *α*‐melittin‐NPs restricted CD8^+^ T cell proliferation in the ACD model, we further investigated whether *α*‐melittin‐NPs mediated immune suppression by restricting CD4^+^ T cell differentiation. Here, we collected the dLNs of *α*‐melittin‐NP‐ and PBS‐treated AD model mice and analyzed the immune function of CD4^+^ or CD8^+^ T cells. As shown in Figure S13 (Supporting Information), the release of IFN‐*γ* in CD8^+^ T cells and IL‐4 in CD4^+^ T cells were both significantly inhibited by *α*‐melittin‐NP and melittin treatments. This result further indicated that T cell activation was inhibited by *α*‐melittin‐NPs in the dLN. Additionally, we explored the effect of *α*‐melittin‐NPs on T cell homing to skin in the AD model by assessing CLA‐ and CXCR3‐positive T cells in blood and lymph nodes. Our data showed that CLA and CXCR3 levels were not substantially affected by *α*‐melittin‐NP treatment (Figure S14, Supporting Information).

Furthermore, under *α*‐melittin‐NP treatment, the concentration of IL‐4 in the AD lesions decreased by 91.9% (Figure 5F), the concentration of IL‐6 decreased by 59.7% (Figure 5G), and the concentration of TNF‐*α* decreased by 40.8% (Figure 5H). We also observed similar decreases in the concentrations of IL‐4, IL‐6, and TNF‐*α* with melittin treatment or DEX treatment. These data indicate that *α*‐melittin‐NP treatment effectively controls the activation of Th2 cells with the associated release of IL‐4, IL‐6, and TNF‐*α*. During the development of AD, thymic stromal lymphopoietin (TSLP) released by epidermal cells induces the strong differentiation of Th2 cells by stimulating DC activation.^[^ ^35^ ^]^ In our OXA‐induced AD‐like model, the TSLP expression in *α*‐melittin‐NP‐ treated group decreased by 79.5% and was not significantly different from that in untreated normal skin (Figure 5K).

Furthermore, we detected the IgE and IgM expression in the spleen, and monitored the T cells and B cells distribution in the spleen and to investigate whether *α*‐melittin‐NPs induce immunity change in the spleen. As shown in Figure S15 (Supporting Information), the IgE and IgM in the spleen were significantly suppressed in *α*‐melittin‐NP‐treated AD mice when compared with PBS‐treated AD mouse. The data in Figure S16 (Supporting Information) showed that *α*‐melittin‐NPs treatment did not significantly induce the percentage change of B cells, CD4 T cells, and CD8 T cells in the spleen, dLN, blood, and bone marrow. The immunofluorescent section data of the spleen also showed that *α*‐melittin‐NPs treatment did not induce the change in spleen structure (Figure S17, Supporting Information). Thus, *α*‐melittin‐NPs treatment suppressed IgE and IgM expression in the spleen without inducing B cells and T cells percentage change or spleen structure change.

Together, these results indicated that *α*‐melittin‐NPs restrict the release of Th2‐type cytokines (IL‐4, IL‐6, and TNF‐*α*) and TSLP in the AD‐like model.

### 2.7 No Adverse Effects of *α*‐Melittin‐NP Treatment on AD

**Figure 6:**
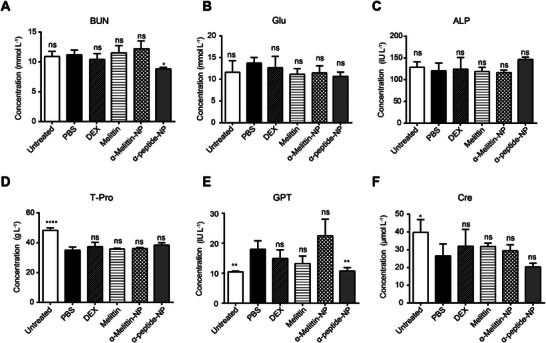
*α*‐Melittin‐NPs had no adverse effects on AD mice. A–F) Evaluation of the effects of *α*‐melittin‐NPs, melittin, *α*‐peptide‐NPs, or DEX on liver and kidney metabolism in AD model mice (*n* = 4 mice per group). A) Quantification of the serum BUN level. B) Quantification of the serum GLU level. C) Quantification of the serum ALP level. D) Quantification of the serum total protein level. E) Quantification of the serum GPT level. F) Quantification of the serum Cre level. Error bars indicate SD; *p < 0.05, **p < 0.01, ***p < 0.001, ****p < 0.0001, and ns, not significant. Statistical analysis was performed using one‐way ANOVA followed by Bonferroni's post hoc test.

To monitor whether *α*‐melittin‐NP, *α*‐peptide‐NP, melittin, or DEX administration induces adverse effects in the AD‐like model, AD mice were administered different treatments, and the biochemical effects of the treatments were assessed (**Figure** **6**). Untreated mice and AD mice treated with intradermal PBS injection (PBS‐AD) were both used as controls. Compared to PBS‐AD mice, *α*‐melittin‐NP‐treated mice did not show any measurable adverse effects on liver function (GLU, 11.5 ± 1.6 mmol L^−1^; ALP, 116.3 ± 5.6 IU L^−1^; T‐PRO, 36.0 ± 0.8 g L^−1^; and GPT, 22.5 ± 5.6 IU L^−1^) or renal function (BUN, 12.2 ± 1.4 mmol L^−1^; CRE, 29.2 ± 3.4 µmol L^−1^). In addition, no significant difference in histology was observed among the untreated, PBS‐AD, *α*‐melittin‐NP, *α*‐melittin‐NP, melittin and DEX groups. Harvested livers and kidneys did not show any significant pathological abnormalities (Figure S18, Supporting Information). Additionally, the treatment of ACD and AD through the intradermal injection of *α*‐melittin‐NPs did not cause skin thinning. Thus, these data confirm that the strategy of continuous intradermal *α*‐melittin‐NP administration does not cause measurable adverse effects.

## 3 Discussion

Exploration of the nanoimmunomodulatory approach targeting the immune system is promising to achieve greater therapeutic effects on treating T cell‐mediated skin disorders while avoiding the side effects of corticosteroid administration. Here, we developed a powerful nanoimmunotherapy approach based on DC‐targeted *α*‐melittin‐NPs for treating ACD and AD.

Consistently, local application of immunosuppressants (such as corticosteroids) is the preferred treatment for chronic ACD and AD. However, the immune response of ACD and AD does not exclusively occur in skin lesions. Soluble antigens, pro‐inflammatory cytokines, such as TSLP, and antigen‐loaded DCs quickly infiltrate LNs, inducing the activation and proliferation of antigen‐specific effector and memory T cells in LNs and resulting in aggravated disease during the pathogenesis of ACD and AD. Thus, the delivery of immunomodulatory drugs targeting LNs and regulating T cell activation and proliferation might provide a new approach for the treatment of ACD and AD. Here, the *α*‐peptide‐NP scaffold significantly enhances the therapeutic effects of melittin on treating ACD and AD, and the enhanced effects are closely related to the fact that *α*‐peptide‐NPs highly target and deliver melittin into the LNs and efficiently suppress allergen‐induced DC and macrophage activation in the LN. Thus, our intradermal injection of *α*‐melittin‐NPs enabled the treatment efficacy in T cell‐mediated skin disorders to not be restricted to the injection region.

DCs are the most important APCs that maintain the dynamic balance between immunity and tolerance. Inhibition of costimulatory molecules on DCs has been verified to strongly inhibit stable DC‐T cell interactions, which profoundly suppress both Th1‐ and Th2‐driven immunity.^[^ ^12^, ^36^, ^37^, ^38^ ^]^ Based on our results, an intradermal injection of a low dose of *α*‐melittin‐NPs (175 nmol per kilogram of body weight) efficiently reduced the proportion of CD80^+^CD86^+^ DCs in the dLNs. In vitro data further revealed that *α*‐melittin‐NPs inhibited the allergen‐ or LPS‐induced differentiation of CD80^+^CD86^+^ BMDCs. In particular, RelB activation in the NF‐*κ*B signaling pathway is critical for the upregulation of costimulatory molecules (CD80 and CD86) on DCs and the subsequent induction of T cell responses via antigen presentation.^[^ ^31^, ^39^ ^]^ Although melittin has been reported to inhibit the NF‐*κ*B, AP1 and MAPK signaling pathways in chondrocytes^[^ ^21^ ^]^ and keratinocytes,^[^ ^40^ ^]^ the regulatory effect of melittin on DC activation and maturation has not been reported. In vitro and in vivo, disruption of RelB expression radically alters DC maturation and inhibits antigen‐specific T‐cell responses.^[^ ^41^ ^]^ RelB phosphorylation is the key molecular event in the RelB‐mediated noncanonical NF‐*κ*B pathway in DCs. Activated cytoplasmic RelB then migrates to the nucleus, initiating the transcription of DC maturation‐related genes (e.g., CD80, CD86).^[^ ^39^ ^]^ Here, our results confirmed that melittin and *α*‐melittin‐NPs efficiently blocked LPS‐induced RelB phosphorylation in a dose‐dependent manner and further inhibited CD80 and CD86 expression in DCs. One limitation of this study is that we mainly detect the effect of *α*‐melittin‐NPs on the NF‐*κ*B pathway by monitoring Relb phosphorylation (Figure 3). In the future, more effort should be performed to address the detailed mechanism of how *α*‐melittin‐NPs inhibit DC maturation via the NF‐*κ*B pathway. As shown in our previous study, immature DCs mainly took up *α*‐peptide‐NPs through nonspecific endocytosis, and mature DCs mainly took up *α*‐peptide‐NPs through the scavenger receptor class B type I (SR‐B1)‐mediated pathway.^[^ ^42^ ^]^ We speculated that both the nonspecific endocytosis‐ and SR‐B1‐mediated pathways participated in the uptake mechanism of the melittin‐NPs in DCs in an inflammatory context, which might account for the no significant difference in phagocytotic capabilities in DCs between inflammatory and noninflammatory contexts. Accumulated melittin in DCs restrained RelB activity in the NF‐*κ*B signaling pathway, which further suppressed DC activation and maturation.

Additionally, our results also confirmed that *α*‐melittin‐NPs suppressed T cell proliferation and activation with restricted IFN‐*γ* secretion in Th1 cells in the ACD model and restricted IL‐4, IL‐6, and TNF‐*α* secretion in Th2 cells in the AD model. Together, these results revealed that *α*‐melittin‐NPs strongly modulated the costimulatory pathway in Th1‐ and Th2‐driven immunity by regulating the costimulatory pathway in DCs. In addition, IgE Abs secreted by B cells is the most important immunoglobulin triggering allergic reaction,^[^ ^43^ ^]^ in which allergen‐specific IgE Abs bind to surface receptors on mast cells and eosinophils and cross‐links with antigen, causing the secretion of inflammatory mediators.^[^ ^44^ ^]^ More and more research reveals that Th2‐released IL‐4 is critical for the induction of IgE^+^ B cells in the spleen.^[^ ^45^ ^]^ Our data in Figure 5E; and Figure S15 (Supporting Information) show that *α*‐melittin‐NP treatment efficiently controlled the serum IgE level and IgE expression in spleen. This result suggests that the *α*‐melittin‐NPs treatment might suppress the induction of IgE^+^ B cells by restricted Th2‐type cytokine release in the AD‐like model. Interestingly, the intratumoral injection of a high dose of *α*‐melittin‐NPs (1750 nmol per kilogram of body weight) exhibits a much higher antitumor activity than an injection of melittin, which not only directly kills tumor cells but also activates the antitumor immune response.^[^ ^46^ ^]^ The therapeutic effect of *α*‐melittin‐NPs may be similar to that of the alkylating agent cyclophosphamide, which exerts strong immunosuppression at a low dose and directly kills tumor cells to stimulate M*φ* activation at a high dose.^[^ ^47^ ^]^ The mechanism that results in the opposing modulation of the immune response induced by different doses of *α*‐melittin‐NPs requires further investigation.

Compared with the complex composition of bee venom therapy,^[^ ^18^ ^]^ *α*‐melittin‐NPs are assembled from phospholipids, *α*‐melittin peptides, and cholesterol, with simple and clear ingredients. Compared with an intradermal injection of melittin or topical smearing of melittin, intradermal injection of *α*‐melittin‐NPs avoids the tissue damage caused by local administration of melittin and increases the penetration of melittin. In the AD model (Figure 5E), our data confirmed that *α*‐melittin‐NP treatment efficiently controlled the serum IgE level, while this decrease was not observed in melittin treatment. *α*‐Melittin‐NPs administration also achieved a treatment efficiency of 82.1%, while melittin administration achieved a treatment efficiency of 63.6% in the AD‐like model. Thus, *α*‐melittin‐NPs not only shield melittin‐induced skin damage but also achieved better treatment efficiency than free melittin alone in the AD model. In addition, the administration of melittin‐loaded NPs might achieve a better sustained‐release effect than the administration of melittin alone and might be more appropriate for the continuous treatment of AD. In the future, a combination of *α*‐melittin‐NPs with microneedle therapy might be a more suitable application for clinical treatment, which reduces the frequency of drug administration and lessens the local injury caused by direct dermal injection. It should be noted that AD is a highly diverse repertoire with different T cell subset activation and commensal skin microbiota dysbiosis. As the OXA‐induced AD‐like model was mainly mediated by Th2 cells, our strategy of intradermal delivery of *α*‐melittin‐NPs is restricted to treating AD that is skewed toward a Th2 cell‐mediated immune response. The therapeutic effect of *α*‐melittin‐NPs on other types of AD requires further investigation.

In conclusion, the intradermal injection of low‐dose *α*‐melittin‐NPs suppressed allergen‐stimulated DC activation and further inhibited allergen‐specific T cell proliferation and activation, leading to efficient control of both the Th1 and Th2 cell‐driven immune responses in ACD and AD. Intradermal delivery of low‐dose *α*‐melittin‐NPs not only shields melittin‐induced skin damage but also efficiently elicits immunosuppression against T cell‐mediated cutaneous immunity. Our approach of loading melittin into toxicity‐protective lipid nanoparticles and targeting LNs provides new and promising immunoregulatory therapeutic to treat ACD and AD.

## 4 Experimental Section

### Reagents

RPMI‐1640 medium was purchased from HyClone (Logan, USA). Fetal bovine serum (FBS), 2‐mercaptoethanol (2‐ME), L‐glutamine, nonessential amino acids, sodium pyruvate, penicillin, and streptomycin were purchased from Invitrogen Gibco (BRL, USA). 4‐(2‐Hydroxyethyl)‐1‐piperazineethanesulfonic acid (HEPES), cholesteryl oleate, OXA, DNFB, and DNBS were purchased from Sigma‐Aldrich Co. (St. Louis, MO). 1,2‐Dimyristoyl‐sn‐glycero‐3‐phosphocholine (DMPC) was obtained from Avanti Polar Lipids Inc. (Alabaster, AL). *α*‐Peptide (DWFKAFYDKVAEKFKEAF‐NH_2_) and *α*‐melittin (DWFKAFYDKVAEKFKEAF‐GSG‐GIGAVLKVLTTGLPALISW‐IKRKRQQ‐NH_2_) peptides were synthesized by Apeptide Co., Ltd. (Shanghai, China). Melittin was purchased from Bankpeptide Co., Ltd. (Hefei, China). DEX was purchased from Tianya Jin Kinyork Group Co., Ltd. (Hubei, China).

### Mice

Female wild‐type C57BL/6 mice (6–12 weeks old) were purchased from the Hubei Research Center of Laboratory Animals (Wuhan, Hubei, China) to study the effects of melittin and *α*‐melittin‐NPs on treating ACD and AD. Albino C57BL/6 mice were obtained from The Jackson Laboratory (Bar Harbor, ME) to investigate the distribution of *α*‐melittin‐NPs in mouse organs and to quantify the toxicity of melittin and *α*‐melittin‐NPs. B6.Cg‐Tg (Itgax‐Venus)1Mnz/J (CD11c^Venus^) and C57BL/6‐Tg(TcraTcrb)1100Mjb/J (OT‐I) transgenic mice were obtained from The Jackson Laboratory (Bar Harbor, ME). All animals were bred and maintained in a specific pathogen‐free barrier facility at the Animal Center of Wuhan National Laboratory for Optoelectronics and used at 6–12 weeks of age. All animal studies were approved by the Hubei Provincial Animal Care and Use Committee (2019S2044) and were performed in accordance with the experimental guidelines of the Animal Experimentation Ethics Committee of Huazhong University of Science and Technology (Hubei, China).

### Statistical Analysis

All studies were evaluated in at least three independent experiments for each condition to ensure reproducibility. Data are expressed as scatter plots with the mean ± SD or mean± SEM . Significant differences between different groups were determined using two‐tailed unpaired Student's *t*‐tests for two‐group comparisons and one way analysis of variance (ANOVA) with Bonferroni's post hoc test for multiple group comparisons. Statistical analyses were performed using GraphPad Prism software. Differences were considered significant at *p < 0.05, **p < 0.01, ***p < 0.001, and ****p < 0.0001.

Other methods are described in the Supporting Information.

## Conflict of Interest

The authors declare no conflict of interest.

## Author Contributions

Z.L. and Z.F. contributed equally to this work. Z.L. and Z.H.Z. initiated and designed the project. Z.L., Z.F., M.L.X., Y.F.L., J.X.L., and C.L.H., performed the experiments. Z.L. and Z.F. assisted with data processing. Z.L., Z.F., and Z.H.Z. analyzed the data and wrote the paper.

## Supporting information

Supporting Information

## Data Availability

The data that support the findings of this study are available in the Supporting Information of this article.
