# Supplementary material for: Melittin‐Carrying Nanoparticle Suppress T Cell‐Driven Immunity in a Murine Allergic Dermatitis Model
Source: Adv Sci (Weinh). 2023 Jan 13;10(7):2204184. doi: 10.1002/advs.202204184 (PMC9982551; doi:10.1002/advs.202204184)
Supplement: Supplementary file 1 — Supporting Information [file ADVS-10-2204184-s001.pdf]

ADVANCED SCIENCE
Open Access

Supporting Information

for *Adv. Sci.*, DOI 10.1002/advs.202204184

Melittin-Carrying Nanoparticle Suppress T Cell-Driven Immunity in a Murine Allergic Dermatitis Model

*Zheng Liu, Zhan Fan, Jinxin Liu, Jialu Wang, Mengli Xu, Xinlin Li, Yilun Xu, Yafang Lu, Chenlu Han and Zhihong Zhang**

Supporting Information

Title Melittin-carrying nanoparticle suppress T cell-driven immunity in a murine allergic dermatitis model

*Zheng Liu, Zhan Fan, Jinxin Liu, Jialu Wang, Mengli Xu, Xinlin Li, Yilun Xu, Yafang Lu, Chenlu Han, Zhihong Zhang**

Supporting Methods

Preparation of α-melittin-NPs and α-peptide-NPs: The preparation of α-melittin-NPs was described previously.[1] α-Melittin is a hybrid peptide in which the melittin peptide is linked with the C-terminus of another α-helical peptide (DWFKAFYDKVAEKFKEAF-NH2) and the sequence of α-melittin is DWFKAFYDKVAEKFKEAF-GSG-GIGAVLKVLTTGLPALISWIKRKRQQ-NH2. Briefly, DMPC (3 μmol) and CO (0.2 μmol) were mixed with chloroform and dried under nitrogen (NO) to form a uniform lipid film. PBS was added, vortexed with the lipid film and then sonicated for one hour at 48 °C to form a lipid emulsion. Then, α-melittin (D4F-melittin) or α-peptide (D4F) was added dropwise into the lipid emulsion and incubated overnight at 4 °C. Nanoparticles that were core loaded with DiR-BOA were prepared by first mixing DMPC and CO with DiR-BOA to load the fluorescent dye, and the remaining steps were the same as those described above. NPs were purified using a fast protein liquid chromatography system (General Electric Healthcare, NY, USA) and concentrated to the desired concentrations. α-melittin-NP was labeled with FITC in a 0.1 mol L^{-1} Na_2CO_3 solution to study the distribution of NPs in cells. Concentrations were calculated from the peptide concentrations, which were measured in the aqueous phase of the solution extracted from the NPs using a CBQCA protein quantitation kit (cat. no. MP 0667, Invitrogen Corporation, CA, USA). The particle size of the nanoparticles is 14.9 ± 4.2 nm (measured by dynamic light scattering) and the surface charge of α-melittin-NPs was neutral (2.45 ± 0.56 mV).[1]

Sensitization and elicitation of the ACD model: Mice were sensitized with 50 μL of 2% (w/v) OXA diluted in vehicle (acetone: olive oil, 4:1) after the abdomen was shaved. Mice were challenged on day 5 with 10 μL of 0.3% (w/v) OXA on both the dorsal and ventral sides of the ears. The increased thickness of the ears was measured with a dial micrometer (Mitutoyo, Tokyo, Japan). We investigated the immunoregulatory efficacy of melittin by intradermally injecting 0.25, 0.5, or 1 mg kg^{-1} melittin at the tail base. PBS, 0.25 mg kg^{-1}, 0.5 mg kg^{-1} and 1 mg kg^{-1} DEX were also intradermally injected as controls. When exploring the treatment efficacy α-melittin-NP, 176 nmol kg^{-1} melittin (0.5 mg kg^{-1}), 176 nmol kg^{-1} α-melittin-NP (concentration of melittin, 0.5 mg kg^{-1}), or 176 nmol kg^{-1} α-peptide-NP

(concentration of α-peptide, 0.429 mg kg^{-1}) in PBS were intradermally injected in the ACD model. PBS was intradermally injected as control.

Induction of the AD-like mouse model: The AD-like model was induced by sensitization with 2% OXA and multiple challenges with 0.1% OXA using a previously described method with some modifications [2]. OXA challenges were performed on days 5, 7, 9, 11, 13, 15, 17, and 19 after sensitization. In the AD-like model, 176 nmol kg^{-1} melittin (0.5 mg kg^{-1}), 176 nmol/kg α-melittin-NP (concentration of melittin, 0.5 mg kg^{-1}), or 176 nmol kg^{-1} α-peptide-NP (concentration of α-peptide, 0.429 mg kg^{-1}) in PBS were intradermally injected. PBS or 2548 nmol kg^{-1} (1 mg kg^{-1}) DEX were intradermally injected as controls. Ear thickness was measured with a dial micrometer. The increase in ear thickness was calculated as follows: (ear thickness after OXA application – ear thickness before OXA application) – (ear thickness after vehicle application – ear thickness before vehicle application).

Culture of BMDCs and BMDMs: C57BL/6 mice were sacrificed, and the femurs and tibias were dissected using scissors. The bones were flushed with a syringe filled with RPMI-1640 medium to isolate Bone Marrow (BM) cells. BM cells were cultured in RPMI-1640 medium supplemented with 10% FBS, 100 U mL^{-1} penicillin–streptomycin, 50 μmol L^{-1} β-mercaptoethanol, 20 ng mL^{-1} murine granulocyte-macrophage colony-stimulating factor (GM-CSF) and 1 ng mL^{-1} IL-4 (PeproTech). On day 3 and day 6, half of the supernatant was gently removed and replaced with the same volume of the medium. BMDCs were obtained by collecting nonadherent cells and purified with 30% Percoll (Sigma–Aldrich) to achieve the best viability on day 9. BMDCs were further verified by evaluating the expression of CD11c using flow cytometry. Attached cells were collected by washing with ice-cold PBS and incubating them for 30 min on ice to obtain BMDMs. BMDMs were further verified by confirming high expression of CD11b and F4/80 using flow cytometry. BMDCs or BMDMs were then seeded in 96-well plates, stimulated with DNBS (10 μg mL^{-1}) or LPS (100 ng mL^{-1}) and cocultured with α-melittin-NPs, α-peptide-NPs or melittin for 24 h. The BMDCs or BMDMs were then harvested, and the expression of the costimulatory molecules CD80 and CD86 was evaluated using flow cytometry. BMDCs were incubated with LPS and each reagent at various doses (1 μmol L^{-1}, 2.5 μmol L^{-1}, or 5 μmol L^{-1}) for 60 min to measure pRelA and pRelB levels. The BMDCs were then pre-stained with anti-mouse antibodies specific for CD11c (BV421, clone N418) and MHCII (Percp5.5, clone M5/114.15.2). The pre-stained BMDCs were then fixed at 4 °C for 20 min (avoiding lights) and subsequently stained with anti-mouse pRelA (Ser536, 93H1) and pRelB (Ser552, D41B9) antibodies according to the manufacturer's instructions (CST).

Investigation of the toxicity of melittin and α-melittin-NPs in vivo: Ten microliters of 0.14, 0.70, or 3.52 nmol melittin or α-melittin-NPs were intradermally injected into one ear, and PBS was injected into the contralateral ear. Ear thickness was measured with a dial micrometer at 12 h after injection. The increase in ear thickness was calculated as follows: (ear thickness after melittin or α-melittin-NP injection – ear thickness before melittin or α-melittin-NP injection) – (ear thickness after PBS injection – ear thickness before PBS injection).

Investigation of the toxicity of melittin, α-melittin-NPs and α-peptide-NPs in vitro: BMDCs were collected on day 9, and 2×10^4 BMDCs were seeded in each well of 96-well plates and incubated at 37 °C with 5% CO_2 for 4 h before the incubation with melittin, α-melittin-NPs and α-peptide-NPs. Melittin, α-melittin-NPs and α-peptide-NPs (0.01, 0.1, 1, 2.5, 5, or 10 μmol L^{-1}) were cultivated with BMDCs for 2 h, and then we used a Cell Counting Kit-8 (CCK-8, MedChemExpress) to detect the viability of BMDCs. Murine mastocytoma cells (P815) were collected, seeded in 96-well plates (1×10^5 cells per well), and then cultivated with different doses (0.01-5 μmol L^{-1}) of α-peptide-NPs, α-melittin-NPs and melittin for 30 min. 159 μmol L^{-1} and 1590 μmol L^{-1} compound 48/80 (0.1 mg mL^{-1} and 1 mg mL^{-1}) was used as a control. Mast cell viability was determined by performing CCK-8 assays according to the manufacturer's instructions. Briefly, 1 μl of CCK-8 reagent was added per 100 μl medium and cells were cultured for an additional 1.5 h. Then, the absorbance at 450 nm was measured using a Gen5 Microplate Reader (BioTek Instruments).

Measurements of degranulation: Degranulation was monitored by measuring the release of histamine in the supernatants. P815 cells were collected and washed twice with PBS by centrifugation at 300 × g for 5 min at 4 °C and resuspended in a high glucose DMEM medium containing 10% heat-inactivated FBS. The cells were then treated with various concentrations (0.01-5 μmol L^{-1}) of α-peptide-NPs, α-melittin-NPs or melittin and incubated for 30 min at 37 °C. The supernatants were then collected to detect the amount of histamine released. An ELISA was performed according to the manufacturer's manual. The O.D. of the samples was detected with a UV–visible absorbance microplate reader at a wavelength of 450 nm.

Lymphocyte proliferation assay and cytokine production: For DNBS-dependent proliferation, mice were sensitized with 25 μL of 0.5% (w/v) DNFB diluted in vehicle (acetone: olive oil, 4:1) on the abdomen on day 0 and day 1. On day 5, the skin-draining axillary LNs of the DNFB-sensitized mice were collected to obtain single-cell suspensions. LN cells were then labeled with CFSE (Molecular Probes, Eugene, USA) for 5 min at 37 °C in PBS. An equal volume of FBS was added to stop the labeling reaction. Cells were then washed with culture medium. CFSE-labeled LN cells (5×10^5) were cultured with 0 or 100 μg mL^{-1} sodium DNBS for 72 h, and 1.9 μmol L^{-1} (10 μg mL^{-1}) α-melittin-NPs were added as indicated. The cells were then cultured in cell plates at 37 °C with 5% CO_2. RPMI-1640 medium containing 10% heat-inactivated FBS, 50 μmol L^{-1} β-mercaptoethanol, 2 mmol L^{-1} L-glutamine, 25 mmol L^{-1} HEPES, 1 mmol L^{-1} nonessential amino acids, 1 mmol L^{-1} sodium pyruvate, 100 U mL^{-1} penicillin, and 100 μg mL^{-1} streptomycin was used as the culture medium. Culture supernatants were collected at 72 h post incubation to measure cytokine production. The concentration of IFN-γ was measured using an ELISA (BioLegend, CA, USA).

In vitro $CD4^+$ and $CD8^+$ T cell proliferation assay: BMDCs were cultured as described above. On day 6, BMDCs were collected, washed extensively and preincubated in 96-well flat-bottom plates at 37 °C for 3 h. The spleen from OT-I mice was isolated and digested with collagenase IV (500 μg ml^{-1}) and DNAse I (50 U mL^{-1}) for 30 min at 37 °C, and then filtered through a 70 μm cell strainer. $CD4^+$ and $CD8^+$ T cells were enriched to >85% purity using magnetic-activated cell sorting (Thermo Fisher). BMDCs (1×10^4 per well) and CFSE-labeled $CD8^+$ T cells (1×10^5 per well) were incubated in a 1:10 ratio in 96-well flat-bottom

plates. Mixed cells were stimulated with/without $OVA_{257-264}$ (SIINFEKL) peptide (0.2 μmol L^{-1}) and incubated with/without α-melittin-NPs (5 μmol L^{-1}) for 72 h at 37 °C. Cell proliferation was determined by measuring the CFSE decay in proliferating $CD8^+$ T cells. To further investigated whether α-melittin-NPs suppressed T cell proliferation by modulating DC maturation, BMDCs were preincubated with α-melittin-NPs or α-peptide-NPs for 24 h with 100 ng mL^{-1} LPS. BMDCs were then washed extensively with PBS to discard nanoparticle residue. α-Melittin-NP-treated BMDCs were then cocultured with purified $CD4^+$ or $CD8^+$ T cells with 200 nmol L^{-1} $OVA_{323-339}$ or $OVA_{257-264}$ for T cell proliferation assay. Cell proliferation was determined by measuring the CFSE decay in proliferating $CD4^+$ or $CD8^+$ T cells.

Flow cytometry: After two washes with PBS containing 2% FBS and 1 mmol L^{-1} EDTA (FACS buffer), LN cell suspensions were incubated with an anti-mouse FcRII/III antibody (2.4G2) for 10 min at 4 °C in FACS buffer and then stained with anti-mouse antibodies specific for CD4 (Alexa Fluor 647, clone GK1.5) and CD8 (PE-Cy7, clone 53-6.7) to detect T cell proliferation. The α-melittin-NP (FITC) distribution and absorption in LN and skin cells were analyzed by staining LN cells with anti-mouse antibodies specific for CD3 (PE, clone 145-2C11), CD19 (PE-Cy7, clone 6D5), CD11b (PE-Cy7, clone M1/70), F4/80 (BV421, clone T45-2342), CD11c (AF647, clone N418), MHC-II (PE, clone M5/114.15.2), CD11b (APC, clone M1/70), CD3 (AF594, clone 17A2), CD4 (BV421, clone 30-F11), CD8 (AF647, clone 53-6.7), CD80 (PE, clone 16-10A1), and CD86 (APC, clone GL-1). Skin cells were stained with anti-mouse CD11c (BV421, clone N418), anti-mouse MHC-II (Percp5.5, clone M5/114.15.2), anti-mouse CD207 (PEcy7, clone 4C7), anti-mouse CD64 (PE, clone X54-517.1), anti-mouse CD11b (AF488, clone M1/70), and anti-mouse Ly-6G (APC, clone 1A8) antibodies. For intracellular staining, cell surface antigen staining was first performed, and cells were fixed with 200 μL per tube Fixation Buffer in the dark for 20 min at room temperature. Then, these cells were washed 3 times, resuspended in intracellular staining perm wash buffer and stained with anti-mouse IFN-γ (PE, clone XMG1.2) and anti-mouse IL-4 (APC, clone 11B11) antibodies. APC-conjugated anti-mouse pRelA (Ser536, 93H1) and PE-conjugated anti-mouse pRelB (Ser552, D41B9) antibodies were used to detect the transcription factors of the NF-κB/Rel family in BMDCs according to the manufacturer's instructions. All antibodies were purchased from BioLegend, BD Bioscience and Cell Signaling Technology (CST). 7-AAD (BioLegend) or dye 780 (Thermo Fisher) was used to exclude dead cells. Flow cytometry was performed using a Guava InCyte analyzer (Guava Technologies, CA, USA) and a CytoFLEX flow cytometer (Beckman Coulter, USA). Data were analyzed using FlowJo software (FlowJo, Ashland, OR, USA).

Immunofluorescence staining: For analysis of the α-melittin-NP distribution in LN sections, freshly harvested inguinal LNs were rinsed with PBS and fixed with 4% PFA for 4 h at 4 °C. For analysis of the T and B cells distribution in spleen sections, freshly harvested spleens were rinsed with PBS and fixed with 4% PFA for 12 h at 4 °C. Then, the fixed LNs and spleens were embedded in ultralow gelling temperature agarose (Sigma–Aldrich, MO, USA). Fifty-micrometer-thick sections were obtained using a Leica VT1200 vibrating blade microtome (Wetzlar, Germany). For staining, LN or spleen sections were washed once with PBS, followed by incubation with staining buffer (1% BSA and 0.2% Triton-X100 (Sigma–Aldrich) in PBS) for 1 h. Sections were then stained with antibodies specific for CD169 (AF594, Clone 3D6.112, 1:100), B220 (AF647, Clone: RA3-6B2, 1:100), CD11c (BV421, Clone N418, 1:100), and CD3 (PE, Clone: 145-2C11, 1:100) in staining buffer overnight. All

LNs sections were imaged with an LSM 710 laser confocal scanning microscope (Zeiss, Germany). All spleens sections were imaged with an UltraVIEW VoX Spinning Disk inverted Confocal Microscope (PerkinElmer, USA). The data were analyzed using Imaris software (Bitplane).

Whole-field fluorescence imaging and intravital imaging: Mice were anesthetized by administering an intraperitoneal injection of 0.2 mg ketamine per gram of body weight and 0.02 mg xylazine per gram of body weight. An in-house whole-field fluorescence imaging system[3] was used to image the α-melittin-NP distribution. A BP 716/40 excitation filter and BP 800/40 emission filter were used for imaging α-melittin-NPs (DiR-BOA). For the intravital imaging of T cells and DCs in dermis. T cells were isolated from OXA-sensitized mice (MACS, Stemcell Tech) and labeled with the cell tracker dye CMTMR (Invitrogen) and intravenously injected into CD11c-Venus mice. All imaging was performed in a field of 424 μm × 424 μm (512 × 512 pixels) with excitation wavelengths of 488 nm for Venus and 561 nm for CMTMR. Images were acquired at a rate of two frames per minute over a z-range of 50 μm (below the epidermis) by performing 10-μm z-steps. More details about the intravital imaging protocol, three-dimensional reconstruction and imaging data analysis refer to our previous work[4].

Distribution of α-melittin-NPs in organs and tissues after injection: FITC-labeled α-melittin-NPs or PBS were intradermally injected into the tail base region of mice in the ACD model. The skin at the injection site and other organs were collected and weighed from the ACD mice at 24 h, 48 h, and 72 h. Next, the tissues were mechanically digested in 1 mL PBS by surgical shears and tissue homogenizer to fully release the FITC signal. The organ tissue suspension after grinding was then centrifuged at 3,000 rpm for 5 min, and 500 μL supernatant was collected for following FITC measurement. Due to the tissue suspension of the liver and brain was extremely thick, the supernatant from the liver and brain was diluted 10 times before the FITC measurement. After that, the supernatant was further centrifugation at 12,000 rpm for 10 min, and 100 μL supernatant was placed in a 96-well plate to detect the FITC emission using a FlexStation 3 multi-mode microplate reader (Molecular Devices, CA, USA).

Cytokine and chemokine quantitation: Total cytokine levels in mouse serum or cell culture supernatant were analyzed using specific ELISA kits (IFN-γ: BioLegend). IgE levels in mouse serum were analyzed with a specific ELISA kit (BioLegend). The local levels of TNF-α, IL-1β, IL-4 and IFN-γ in skin lysates were analyzed with specific ELISA kits (BioLegend). Briefly, an ELISA plate was coated with anti-mouse IFN-γ, IL-4, TNF-α, IL-1β, or IgE monoclonal antibodies (mAbs). The ELISA plates were then washed three times with Tris-buffered saline (TBS) containing 0.05% Tween-20 (TBS-T). Diluted serum samples were added to the wells and incubated for 2 h. After washes with TBS-T, biotinylated anti-mouse IgE was added to each well and incubated for 1 h. After washes with TBS-T, the wells were incubated with avidin-conjugated HRP (BD Bioscience) for 30 min and then washed with TBS-T. The reaction was developed with a substrate chromogen for 30 min and stopped by adding an equal volume of 2 mol L^{-1} H_2SO_4. The optical absorbance at a wavelength of 450 nm was measured. The reaction product of mouse IFN-γ/IgE was used as the standard. The skin was harvested and weighed to measure tissue cytokines in the AD model. Then, tissue samples were minced and lysed in lysis buffer containing PBS and 1% Triton X freshly

supplemented with a protease inhibitor cocktail (Sigma–Aldrich). The lysates were then filtered, aliquoted, and stored at −80 °C until analysis. Samples were assayed using a LEGENDplex^{TM} Mouse Th Cytokine Panel (12 plex, BioLegend) according to the manufacturer's instructions. The data were analyzed using LEGENDplex software (BioLegend).

Quantitative PCR analysis: Total RNA of spleen or ear was extracted using TRIZOL reagent (Invitrogen) and the extraction procedure followed the instructions provided by the manufacturer. The concentration and quality of RNA were quantified by using Nanodrop (Invitrogen). RNA was reverse transcribed into cDNA using the PrimeScript RT Mast Mix kit (TaKaRa) by referring to the protocol provided by the manufacturer. Real-time quantification of PCR was performed by using TB Green Premix Ex TaqTM II (TaKaRa) with QuantStudio 3 Real-Time PCR System (Applied Biosystems, CA) or Applied Biosystems Step one Detector (Applied Biosystems, CA). The sequences of the primers are shown below: IgE forward, gttcgacctgtcaacatcact; IgE reverse, tagaggctagtttgccttcct; IgM forward, gctcagctatgctacgctgt; IgM reverse, tgttctggtagttccaggtgaa; Gapdh forward, aggtcggtgtgaacggatttg; Gapdh reverse, tgtagaccatgtagttgaggtca ; Tslp forward, acggatggggctaacttacaa ; Tslp reverse, agtcctcgatttgctcgaact ; Actin forward, ggctgtattcccctccatcg; Actin reverse, ccagttggtaacaatgccatgt.

Biochemical analyses: Blood samples were collected before the mice were sacrificed and then incubated at 4 °C overnight and centrifuged to obtain mouse serum. Serum biochemical analyses were performed using a biochemical analyzer (SPOTCHEM EZ SP-4430, Arkray Inc., Kyoto, Japan).

Histopathological analyses of skin, liver and kidney in AD models: The skin, livers, and kidneys were extracted from normal or treated mice and fixed in a 4% paraformaldehyde solution. The organs were embedded in paraffin, sectioned, and stained with H&E. The H&E-stained slices were scanned with a Nikon Ni-E (Nikon, Minato, Tokyo, Japan), and images were acquired using NIS-Elements software and further analyzed with ImageJ software.

Supporting Figures and Figure Legends

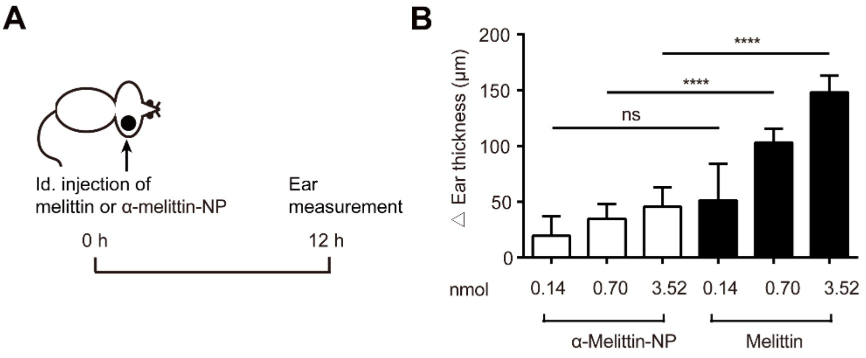

Figure S1. α-Melittin-NP shied the local toxicity *in vivo*. A) Schematic illustrating the quantification of the toxicity of melittin and α-melittin-NP. B) Measurement of ear swelling at 12 h after intradermal injections of different concentrations of melittin or α-melittin-NP into the ears (n = 6 mice per group). Error bars indicate SD, **** $p < 0.0001$ and ns, not significant; unpaired Student's *t test*.

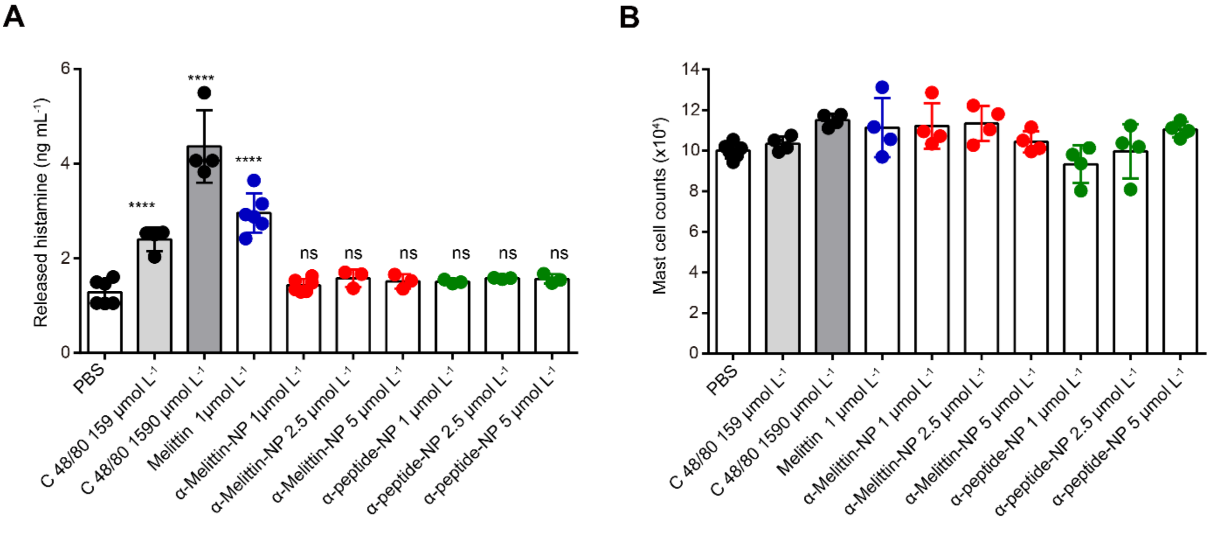

Figure S2. The effect of α-melittin-NPs on mast cell degranulation. A) The amount of histamine released into the supernatant by the murine mastocytoma cell line (P815) cultured with compound 48/80 (C 48/80), melittin, α-peptide-NP, and α-melittin-NP. B) Live mastocytoma cell counts. A total of 100,000 mast cells were stimulated with C 48/80, melittin, α-peptide-NP, and α-melittin-NP for 30 min. n ≥ 3 samples per group, means ± SD, ns, not significant, **** $p < 0.0001$; one-way ANOVA followed by Bonferroni's post hoc test in (A).

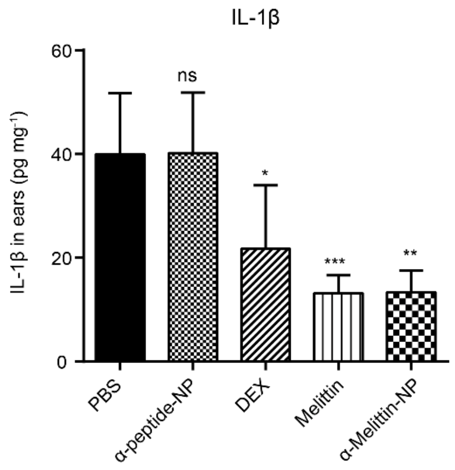

Figure S3. α-Melittin-NP suppressed the local level of IL-1β in the CHS response. Quantification of the IL-1β concentration in each group. The ears from the ACD mice in the PBS-, α-melittin-NP-, DEX-, melittin- and α-peptide-NP-treated groups were harvested (n = 4 mice per group). Total protein was extracted to assess local cytokine levels in inflamed ear tissue. ns, not significant, * $p < 0.05$, ** $p < 0.01$, and *** $p < 0.001$; one-way ANOVA followed by Bonferroni's post hoc test.

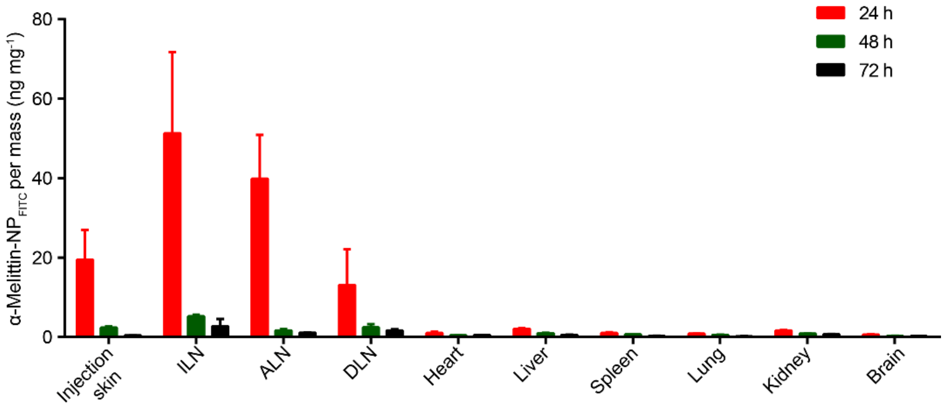

Figure S4. α-Melittin-NPs are mainly restricted to the injected skin and skin-draining LNs (ILN, ALN, DLN). Mice were sensitized with 2% (W/V) OXA in day 0, and challenged with 0.3% (W/V) in day 5. 20 nmol of FITC-α-melittin-NPs (quantification was based on FITC content) were intradermally injected at the tail base 30 min before the rechallenge of OXA. Skins and other organs were collected, weighed, and mechanically digested in PBS for 3 min with an electronic homogenizer. The tissues were centrifuged at 12,000 g for 5 min, and the supernatants were collected to detect FITC content using a FlexStation 3 multi-mode microplate reader (Molecular Devices, CA, USA). N = 3 per group, means ± SEM.

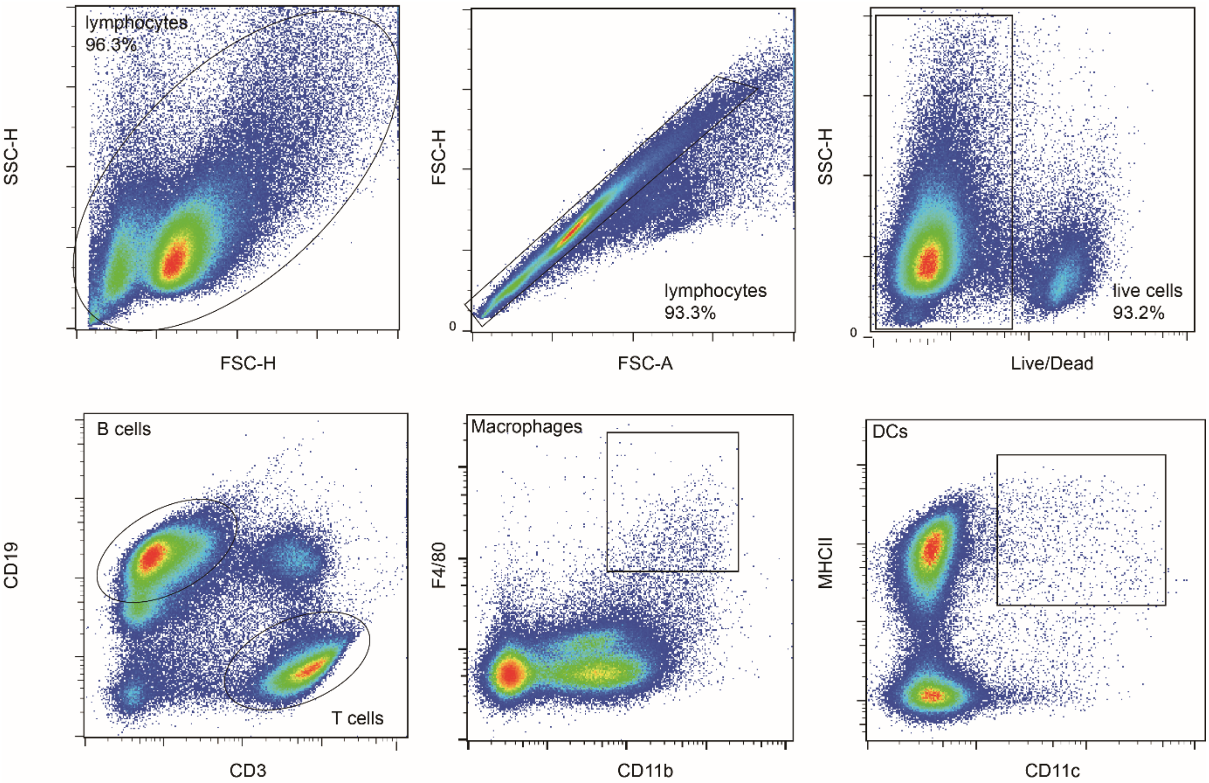

Figure S5. Gating strategy for immune cells in LNs. Gating strategy for immune cells in the LNs at 4 h after intradermal α-melittin-NP (FITC) or PBS injection into the tail base. Cells were gated on single cells and live cells. Live cells were then gated on CD19, CD3, CD11C, MHC-II, CD11b, and F4/80 to define T cells ($CD3^{+}CD19^{-}$), B cells ($CD3^{-}CD19^{+}$), DCs ($CD11c^{+}MHC\text{-}II^{hi}$), and macrophages ($CD11b^{+}$, $F4/80^{+}$). The data are representative of three independent experiments.

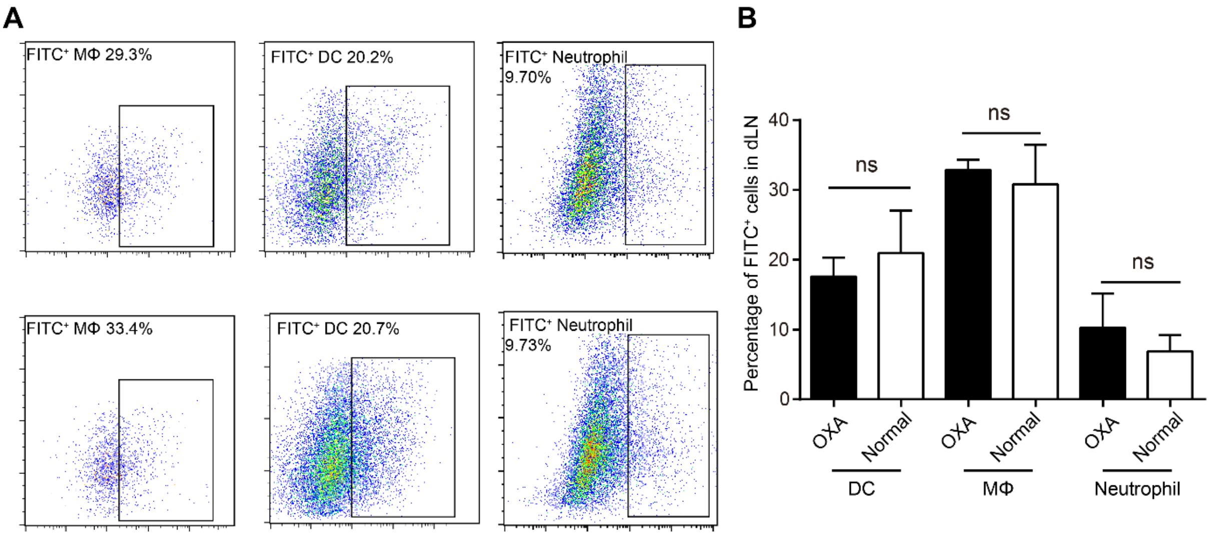

Figure S6. The uptake of α-melittin-NPs in DCs, macrophages, and neutrophils in the OXA-challenged inflammatory context. A). Representative flow cytometry plots of the uptake percentage in macrophages, DCs and neutrophils in the context of OXA-induced inflammation or the untreated state. B) Quantitative statistical analysis of FITC-positive cells in (A). (n ≥ 3 mice per group, mean ± SD). ns, not significant; unpaired Student's *t test* in B.

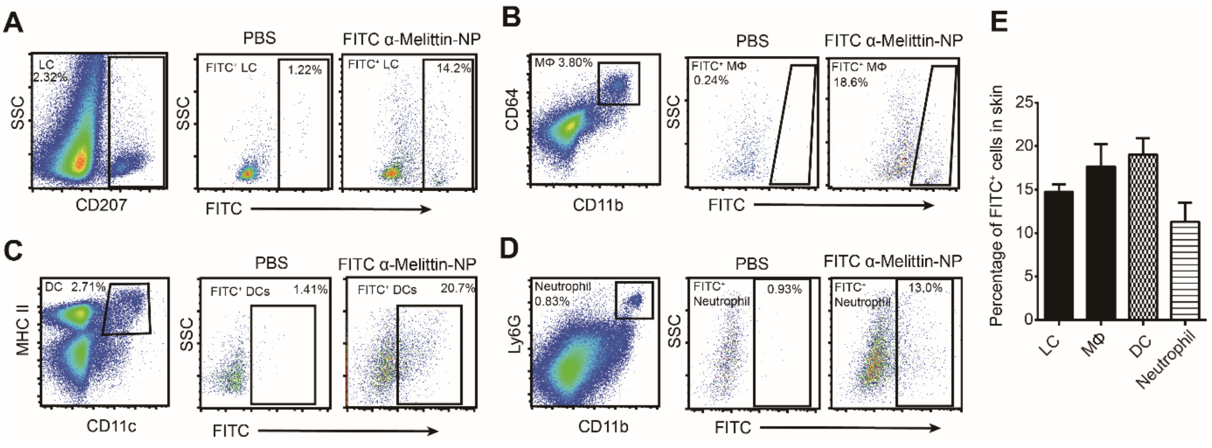

Figure S7. The phagocytotic capacity of LCs and macrophages taking up α-melittin-NPs in the actual challenge response. A-D) Representative flow cytometry plots showing the uptake efficiency of FITC-labeled α-melittin-NPs by LCs (A), macrophages (B), DCs (C) and neutrophils (D) in skin. E) Quantitative analysis of the FITC-positive cells in (A-D). N ≥ 3 mice per group, means ± SD.

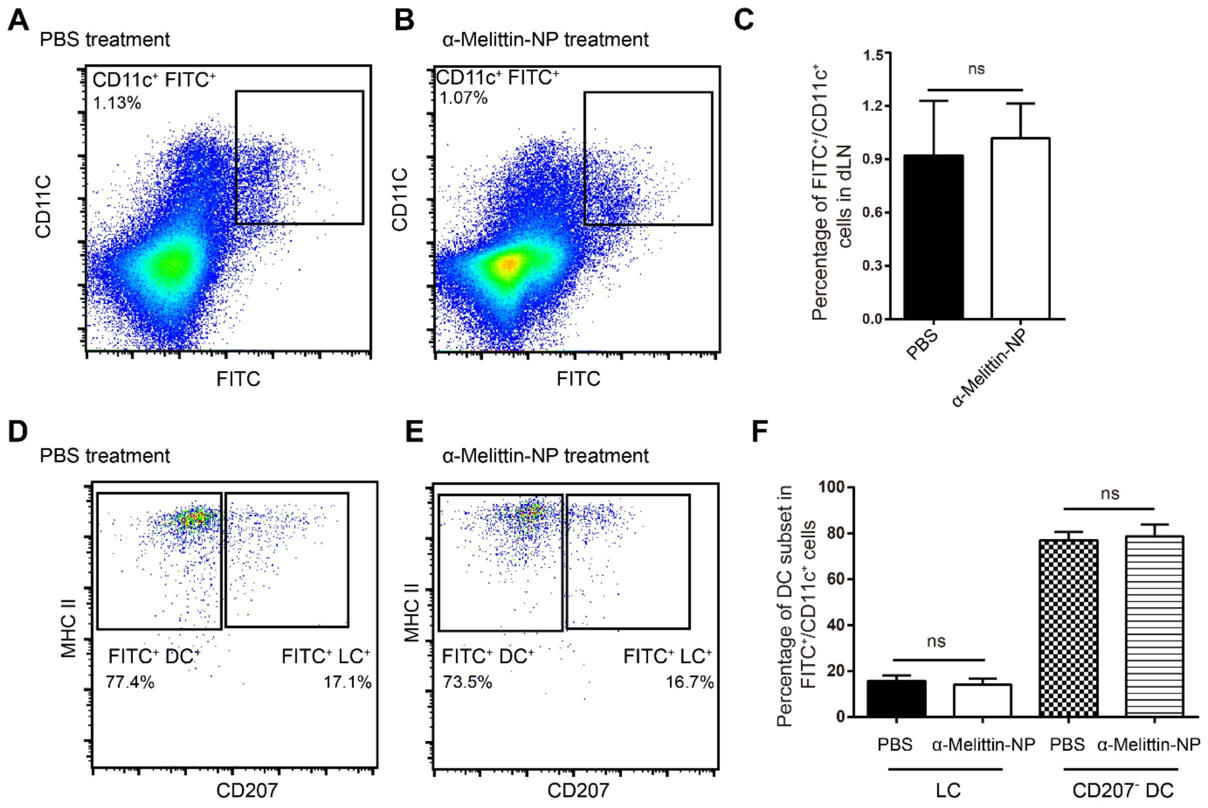

Figure S8. The effect of α-melittin-NPs on skin DC homing in the FITC-challenged model. A-B) The flow cytometry plots of FITC$^+$ DCs cells in LNs from the PBS-treated (A) or α-melittin-NP-treated (B) group. C) Quantitative statistical analysis of FITC$^+$ DCs in (A, B). D-E) Representative flow cytometry data for CD207$^+$ DCs and CD207$^-$ DCs in LNs from the PBS-treated (D) or α-melittin-NP-treated (E) groups. F) Quantitative statistical analysis of FITC$^+$ DCs in (D, E), n ≥ 3 mice per group, means ± SD. ns, not significant; unpaired Student's *t test* in C-F.

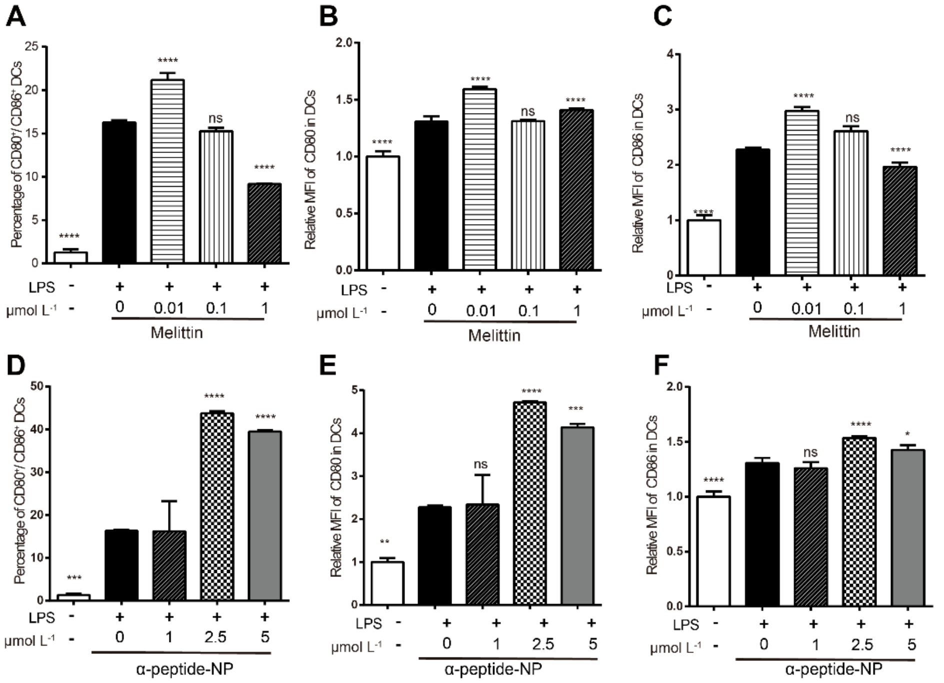

Figure S9. The effect of melittin and α-peptide-NPs on the maturation of BMDCs *in vitro*. A) Quantitative data for the CD80 and CD86 dual-positive DCs in the melittin-treated group. B-C) Quantitative data for the CD80 (B) and CD86 (C) MFIs in LPS-stimulated DCs treated with melittin (n ≥ 3 samples per group, means ± SD). D) Quantitative data for the CD80 and CD86 dual-positive DCs in the α-peptide-NP-treated group. E-F) Quantitative data for the CD80 (E) and CD86 (F) MFIs in LPS-stimulated DCs treated with α-peptide-NPs (n ≥ 3 samples per group, means ± SD). * $p < 0.05$, ** $p < 0.01$, *** $p < 0.001$, and **** $p < 0.0001$; one-way ANOVA followed by Bonferroni's post hoc test in (A-F).

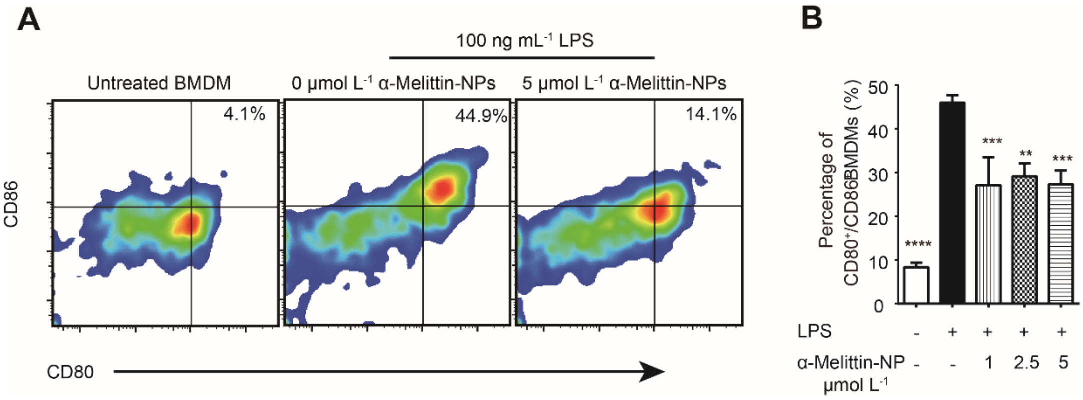

Figure S10. α-Melittin-NPs inhibited BMDM activation. A) Representative flow cytometry plots for the CD80/CD86 dual-positive proportion in BMDMs. B) Histogram showing the quantitative analysis of the CD80 and CD86 dual-positive BMDMs treated with different concentrations of α-melittin-NPs (n ≥ 8 samples per group, means ± SD). The data were pooled from two independent experiments, one-way ANOVA followed by Bonferroni's post hoc test in B.

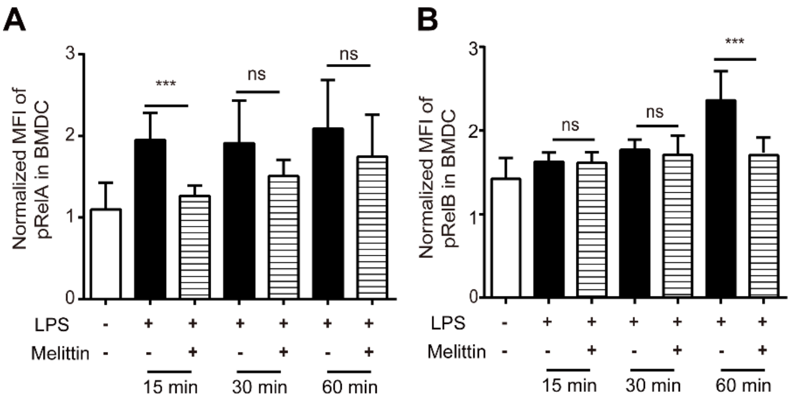

Figure S11. The NF-κB levels in BMDCs with melittin treatment. A-B) Normalized pRelA (A) and pRelB (B) MFIs in BMDCs treated with melittin within 1 h. ns, not significant, and *** $p < 0.001$; unpaired Student's *t test* in A-B. The data were pooled from two or three independent experiments (A-B). N ≥ 6 mice per group, means ± SD.

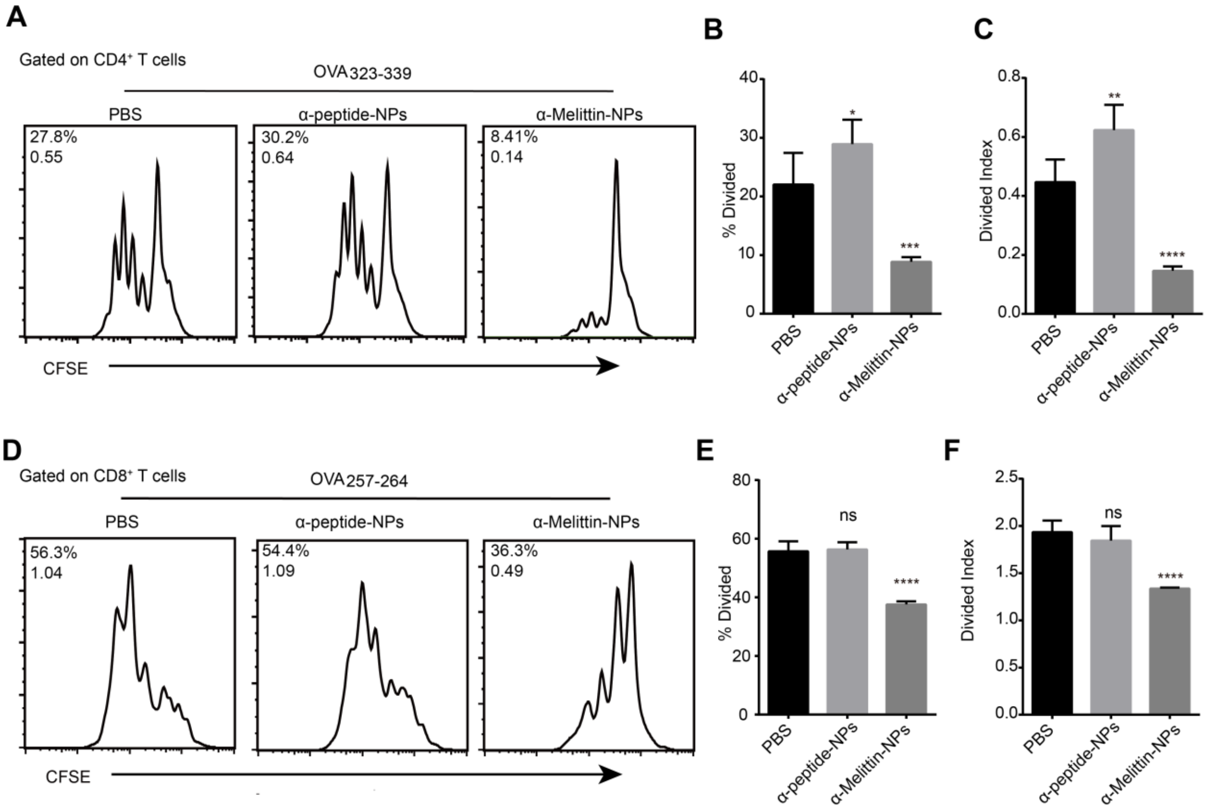

Figure S12. α-Melittin-NPs inhibit antigen-specific T cell proliferation *via* modulating DC maturation. A) Representative flow cytometry plots of $CD4^+$ T cell proliferation induced byα-melittin-NP-, α-peptide-NP- or PBS-treated BMDCs. Numbers in the upper left indicate the percentage of divided cells (% Divided), and numbers in the lower left are the average number of cell divisions (Div. Index). B) Quantification of the percentage of divided $CD4^+$ T cells (% Divided) and C) the average number of cell divisions (Div. Index). ($n \geq 6$ samples per group, means ± SD). D) Representative flow cytometry plots of $CD8^+$ T cell proliferation induced by α-melittin-NP-, α-peptide-NP- or PBS-treated BMDCs. E) Quantification of the percentage of divided $CD8^+$ T cells (% Divided) and F) the average number of cell divisions (Div. Index). $n \geq 8$ samples per group, means ± SD, one-way ANOVA followed by Bonferroni's post hoc test in B-C and E-F.

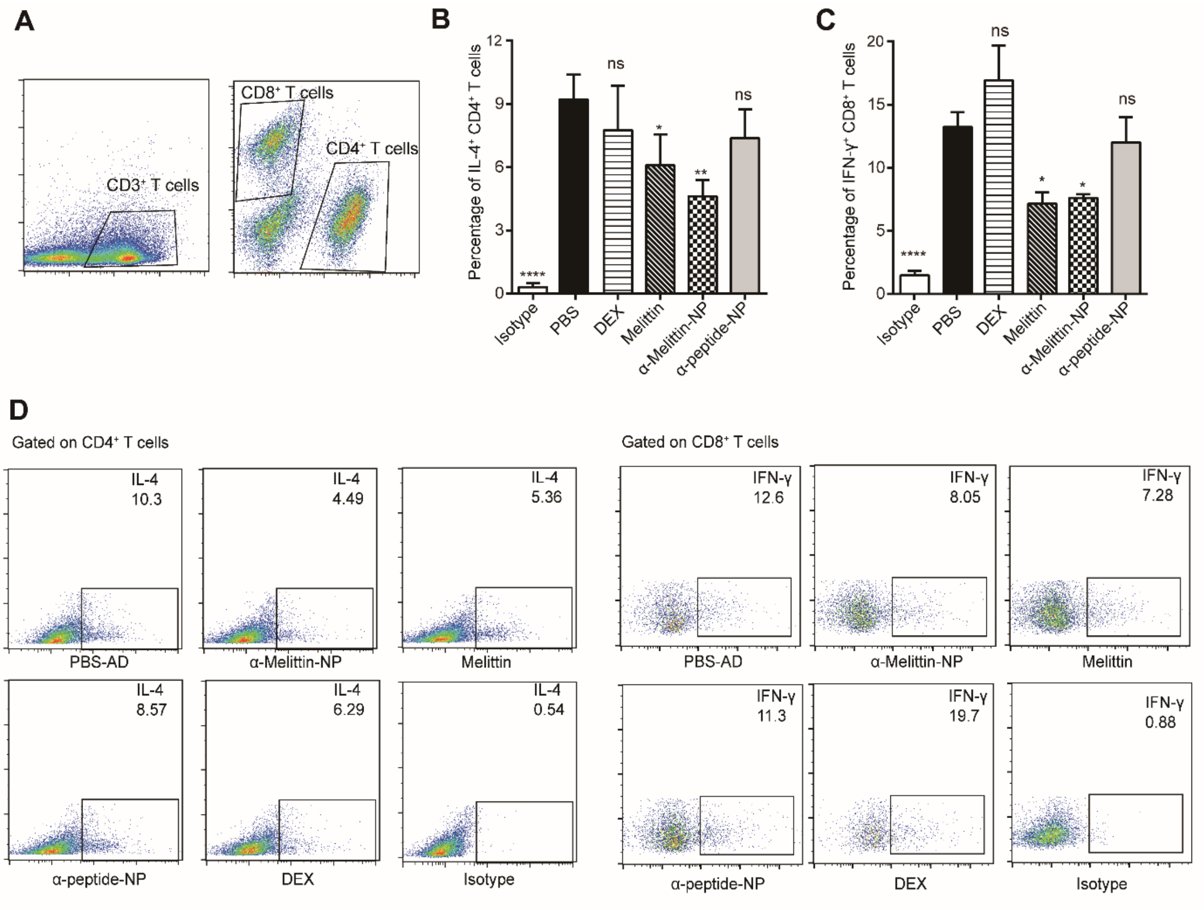

Figure S13. The effect of α-melittin-NPs and melittin on the expression of IFN-γ and IL-4 in T cells from the OXA-induced AD-like model. A) Gating strategy for T cells from the LNs. B-C) Quantitative analysis of IL-4^+ $CD4^+$ T cell and IFN-γ^+ $CD8^+$ T cell subsets in PBS-, DEX-, α-peptide-NP- and α-melittin-NP-treated AD-like models. D) Representative flow cytometry plots of IL-4^+ $CD4^+$ T cells and IFN-γ^+ $CD8^+$ T cells in the dLNs of the PBS-, DEX-, α-peptide-NP- and α-melittin-NP-treated groups. (n ≥ 3 samples per group, means ± SD). * $p < 0.05$, ** $p < 0.01$, and **** $p < 0.0001$; one-way ANOVA followed by Bonferroni's post hoc test in (B-C).

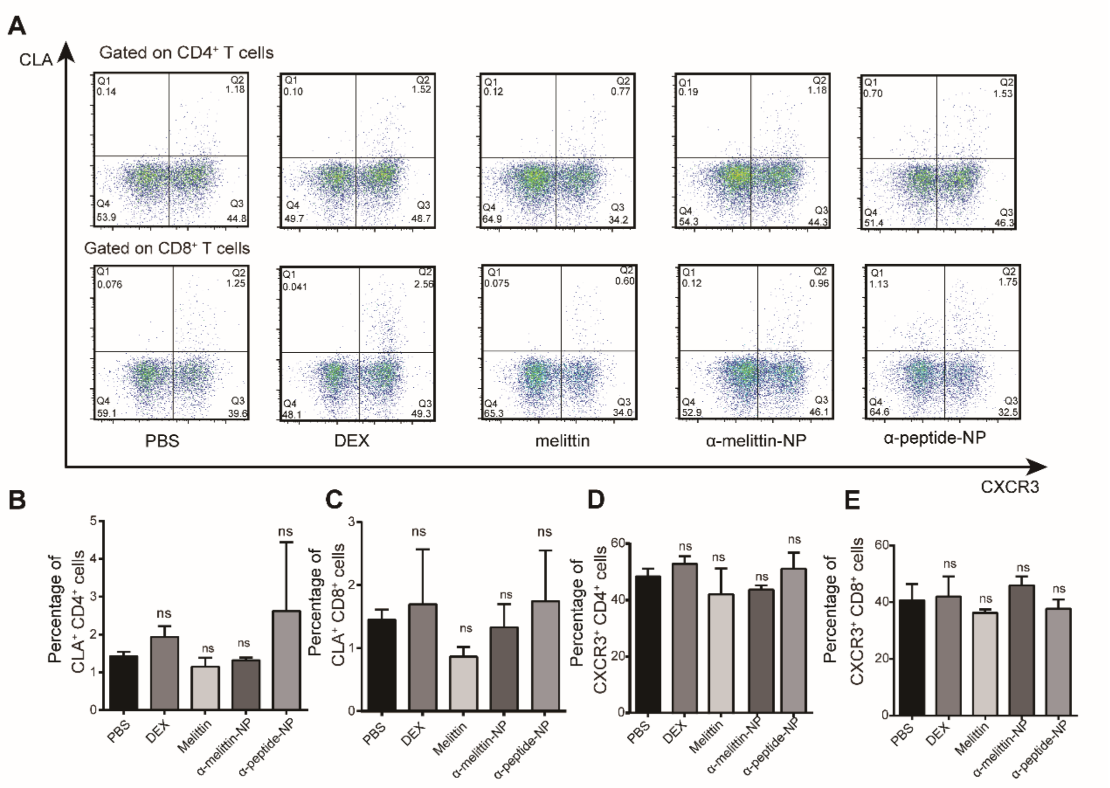

Figure S14. The effect of α-melittin-NPs on T cell homing. A) Representative flow cytometry plots showing the expression of CLA and CXCR3 in skin-homing $CD4^+$ and $CD8^+$ T cells from the PBS-, α-peptide-NP-, DEX-, melittin- and α-melittin-NP-treated AD models. B-E) The quantitative data for CLA- and CXCR3-positive subsets in $CD4^+$ and $CD8^+$ T cells ($n \geq 3$ samples per group, means ± SD). ns, not significant; one-way ANOVA followed by Bonferroni's post hoc test in (B-E).

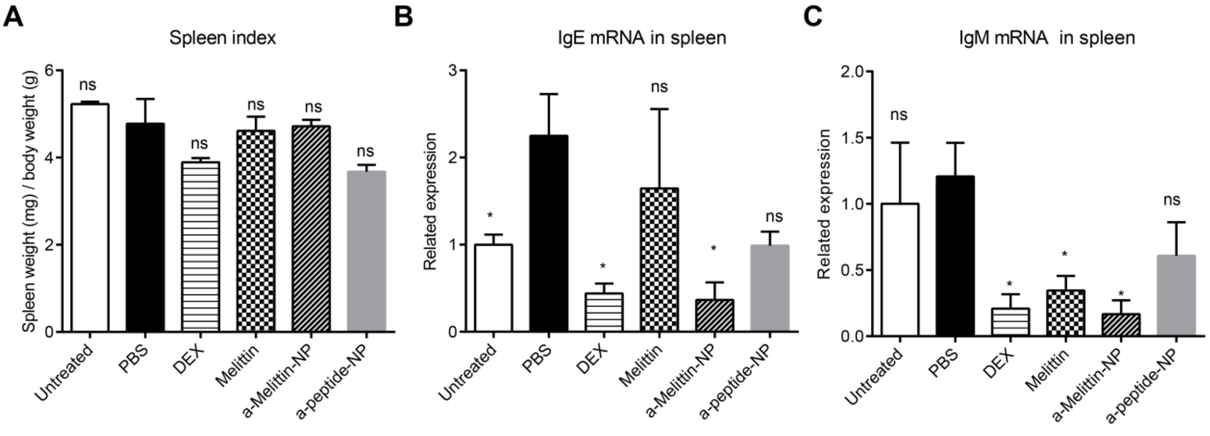

Figure S15. α-Melittin-NPs treatment restricted IgE and IgM expression in spleen. The spleens from AD model mice were collected, and mRNA was extracted to measure the expression of IgE and IgM in PBS, DEX, melittin, α-melittin-NP, and α-peptide-NP groups. A) Spleen index in 5 groups, in which spleen weights were divided by body weights to get the spleen index. B) IgE relative expression in PBS, DEX, melittin, α-melittin-NP, and α-peptide-NP groups ($n \geq 3$ samples per group, means ± SEM). C) IgM relative expression in PBS, DEX, melittin, α-melittin-NP, and α-peptide-NP groups. * $p < 0.05$, one-way ANOVA followed by Bonferroni's post hoc test in (B, C).

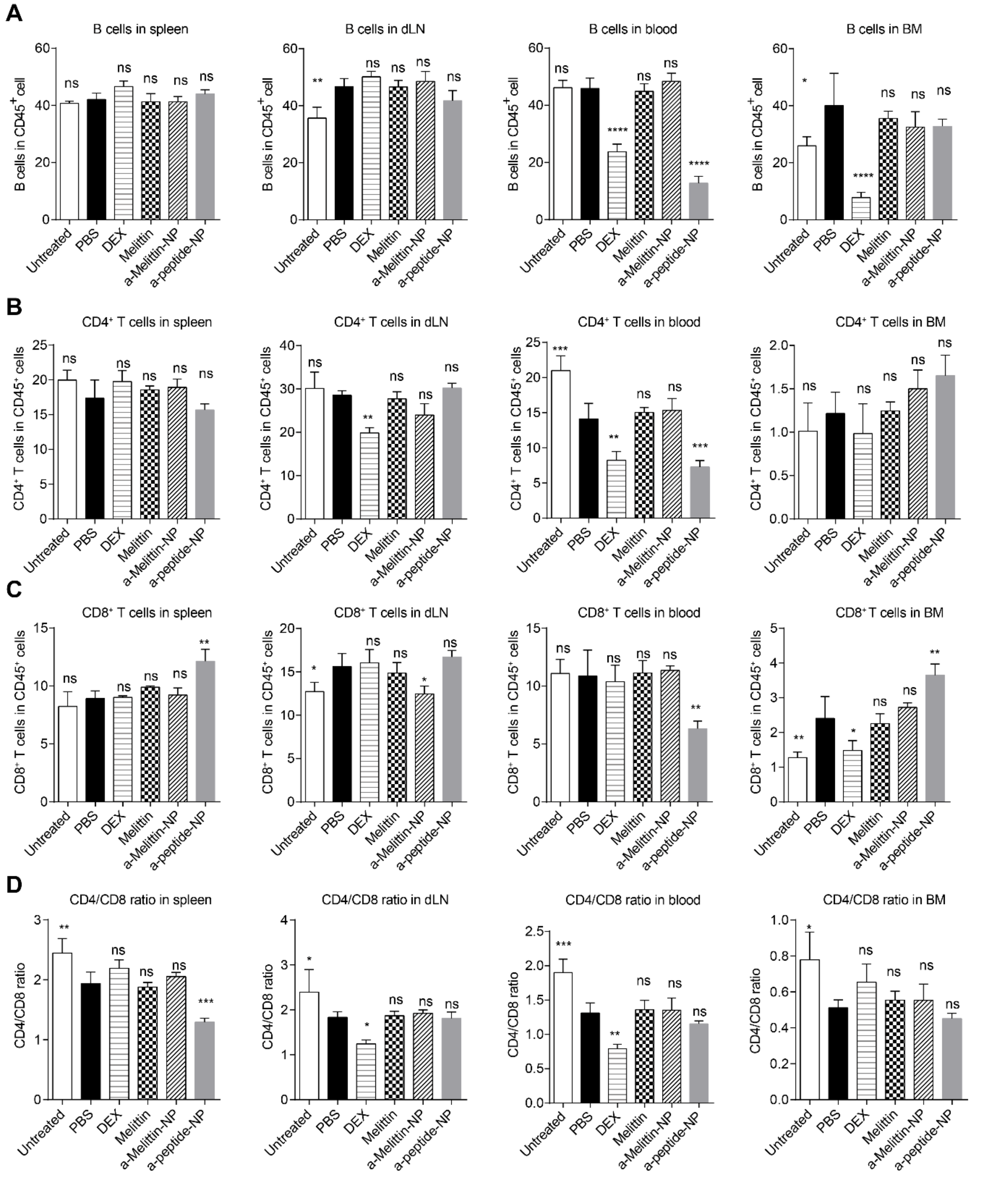

Figure S16. T cell and B cell percentage in different immune organs in AD mice with α-Melittin-NPs treatment. T cell and B cell percentage in different immune organs in AD mice with α-Melittin-NPs treatment (n ≥ 3 samples per group, means ± SEM). The mice of AD models with different treatments were sacrificed and organs were harvested to analyze the T cells and B cells populations. A-C) B cells (A), $CD4^+$ T cells (B), and $CD8^+$ T cells (C) in the spleen, dLN, blood, and BM were analyzed by flow cytometry. D) The histograms display the CD4/CD8 T cells ratio in the spleen, dLN, blood, and BM. * $p < 0.05$, ** $p < 0.01$, *** $p < 0.001$, and **** $p < 0.0001$; one-way ANOVA followed by Bonferroni's post hoc test in (A-D).

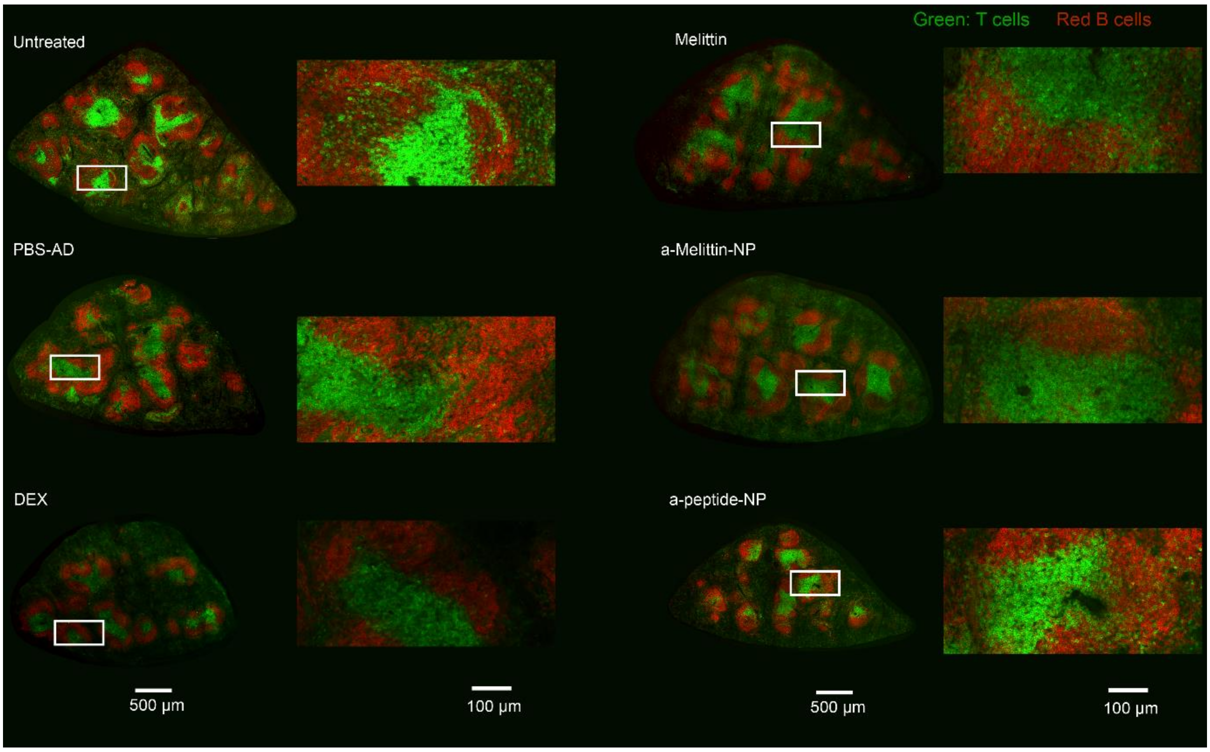

Figure S17. T cell and B cell distribution in the spleen in AD mice with different treatments. The spleen sections of PBS, DEX, melittin, α-melittin-NP and α-peptide-NP groups in AD model are imaged with confocal microscopy. The green color represents $CD3^+$ T cells, and the red color represents $B220^+$ B cells. Scale bars, 500 μm (left panel) and 100 μm (right panel).

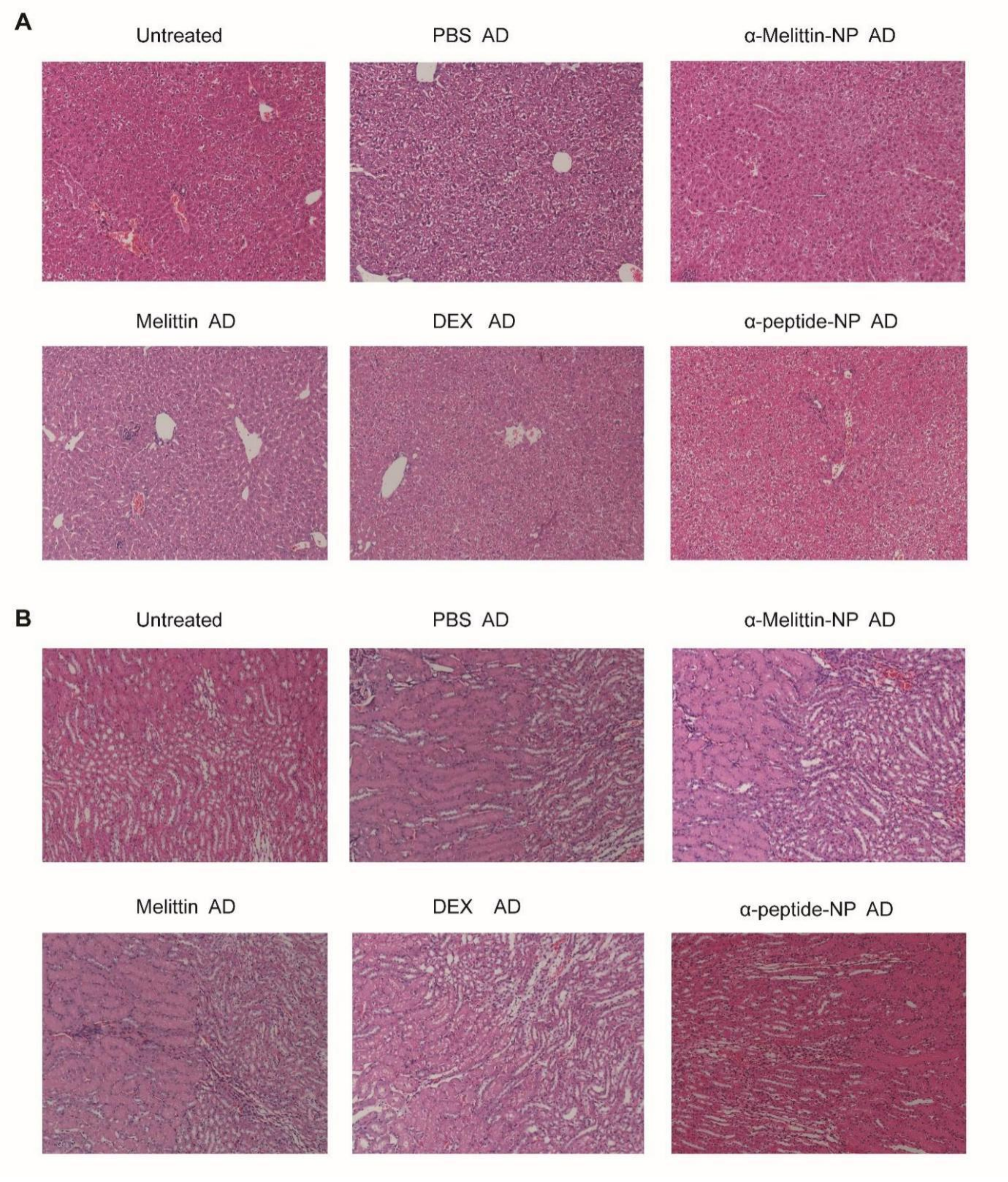

Figure S18. Liver and kidney sections from AD-like mice administered different treatments. Representative images of H&E staining in the livers (A) and kidneys (B) of AD-like mice after PBS, α-melittin-NP, α-peptide-NP, melittin, or DEX treatment. The data are representative of two independent experiments.

References

[1] C. Huang, H. Jin, Y. Qian, S. Qi, H. Luo, Q. Luo, Z. Zhang, *ACS nano* **2013**, *7*, 5791.
[2] M.-Q. Man, Y. Hatano, S. H. Lee, M. Man, S. Chang, K. R. Feingold, D. Y. M. Leung, W. Holleran, Y. Uchida, P. M. Elias, *J. Invest. Dermatol.* **2008**, *128*, 79.
[3] X. Yang, H. Gong, J. Fu, G. Quan, C. Huang, Q. Luo, *Comput. Med. Imaging Graph.* **2012**, *36*, 259.
[4] Z. Liu, F. Yang, H. Zheng, Z. Fan, S. Qiao, L. Liu, J. Tao, Q. Luo, Z. Zhang, *J. Invest. Dermatol.* **2018**, *138*, 1328.
